# Determining the optimal management of geriatric type II odontoid fractures: a comparative network meta-analysis

**DOI:** 10.1007/s10143-026-04170-8

**Published:** 2026-03-07

**Authors:** Shaan Patel, Shiva A. Nischal, Kush M. Kale, Stavros Matsoukas, Joshua Heller, Jack Jallo, James S. Harrop, Srinivas K. Prasad

**Affiliations:** 1 Department of Neurological Surgery, Thomas Jefferson University Hospital, Philadelphia, PA 19107 USA; 2 Department of Physiology, Anatomy & Genetics, Medical Sciences Division, University of Oxford, Oxford, OX1 3PT UK

**Keywords:** Odontoid fracture, Anterior dens screw, Posterior C1-C2 arthrodesis, Non-surgical management

## Abstract

Type II odontoid fractures are the most common cervical injury in geriatric populations, yet optimal management remains debated. This study compared the safety and effectiveness of non-surgical management (NSM), anterior dens screw (ADS) fixation, and posterior arthrodesis (PA) in these patients. A systematic review and frequentist network meta-analysis was conducted according to PRISMA-NMA guidelines. PubMed/MEDLINE, Embase, and CENTRAL were searched from inception to October 2025. Comparative studies including patients ≥ 60 years with Anderson-D’Alonzo type II fractures and reporting at least one primary outcome (mortality, union, stable non-union, unstable non-union) were eligible. Random-effects network models generated pooled odds ratios (ORs) with 95% confidence intervals (CIs). Nineteen studies involving 1242 patients were included (475 NSM; 340 ADS; 427 PA). Mortality did not differ among strategies. PA demonstrated the highest odds of union (OR 8.35, 95% CI 3.79–18.40 versus NSM), followed by ADS (OR 2.19, 95% CI 1.10–4.35 versus NSM). PA also outperformed ADS for fusion (OR 0.26, 95% CI 0.12–0.56). Stable non-union odds were similar across interventions, but unstable non-union was significantly more common after NSM (ADS versus NSM OR 5.93, 95% CI 1.33–26.35; PA versus NSM OR 13.55, 95% CI 2.50–73.36). Systemic morbidity was higher after both ADS (OR 1.87, 95% CI 1.02–3.44) and PA (OR 2.29, 95% CI 1.20–4.36) compared with NSM, while mechanical complications and secondary operation were uncommon and comparable. No global or local inconsistency was detected. In geriatric patients with type II odontoid fractures, PA provides the most reliable fusion and lowest unstable non-union risk, whereas NSM offers comparable survival with lower systemic morbidity but higher structural failure. ADS occupies an intermediate position, benefiting select anatomies where motion preservation or avoidance of prone surgical positioning is advantageous. Treatment should be individualized using a morphology-frailty framework that balances biomechanical stability against physiological reserve.

**Supplementary Information:**

The online version contains supplementary material available at 10.1007/s10143-026-04170-8.

## Introduction

Odontoid fractures (fractures of the C2 vertebral dens) constitute 10–20% of all cervical spine fractures and represent the most common cervical injury in older adults [61]. As global demographic aging accelerates, with adults aged ≥ 60 years projected to comprise ~ 22% of the population by 2050 and ~ 32% by 2100, the incidence, complexity, and downstream burden of geriatric odontoid injury is expected to rise substantially [59]. One-year mortality approaches 30% in this cohort, underscoring the need for treatment strategies that reconcile biomechanical stability with functional preservation and survival [55]. Among the Anderson-D’Alonzo subtypes, fractures occurring at the base of the dens (type II) are both the most prevalent and most biomechanically precarious, marked by high rates of displacement and non-union [3].

Despite their high frequency, the optimal management of odontoid fractures remains contested. Non-surgical management (NSM) using rigid cervical collars or halo vests avoids the physiological stress of anesthesia and operative instrumentation but is associated with higher rates of non-union, persistent pain, and delayed myopathy [8, 62]. Halo vests, in particular, confer additional morbidity including pin-site infection, dysphagia, and pressure-related complications. Conversely, operative fixation strategies demonstrate higher union rates and lower non-union [28, 40, 54], and several observational cohorts have suggested improved short- and intermediate-term survival following surgery [37, 40, 54]. However, surgical gains are not uniform across age strata. Schoenfield et al. (2011) [48] identified differential mortality benefit concentrated in patients aged 65–74, with diminishing returns beyond 85 years. Chapman et al. (2013) [6] similarly noted that although non-operative care was associated with higher early mortality, surgery carried increased perioperative morbidity, longer intensive care unit (ICU) and in-patient stays, and greater gastrostomy dependence. Importantly, these mortality differences are confounded by baseline frailty, whereby higher Charlson Comorbidity Index (CCI) scores are characteristic of patients who die early regardless of treatment modality, suggesting that physiological reserve, rather than operative choice, may be the predominant determinant of survival in geriatric cohorts [37, 58].

Recent systematic reviews and meta-analyses have compared operative and non-operative strategies for geriatric odontoid fractures or contrasted anterior and posterior fixation approaches in isolation [40, 53]. However, these analyses were limited to pairwise comparisons and did not integrate NSM alongside both operative strategies within a unified analytical framework, nor did they leverage network methodology to combine direct and indirect evidence.

Surgical decision-making is further complicated by the divergent physiological profiles of anterior and posterior techniques [22]. Anterior dens screw (ADS) fixation achieves direct fracture compression while preserving C1-C2 rotation, avoiding prone surgical positioning, and limiting vertebral artery manipulation. However, it is highly morphology-dependent and carries a notable risk of dysphagia, reported in up to 35% of elderly patients, with feeding tube dependence and aspiration pneumonia seen in 25% and 19%, respectively [10]. Posterior arthrodesis (PA), including C1-C2 trans-articular or screw-rod fusion techniques, achieves rigid multiplanar stability through tension band restoration and robust fixation, particularly for irreducible or displaced fractures, yet incurs longer operative duration, loss of atlantoaxial rotation, and higher early morbidity. Prior meta-analyses have shown ADS to have lower fusion rates and higher reoperation risk compared to PA, with anterior approaches overall exhibiting greater mechanical complications, non-union, and revision [21, 53]. Crucially, however, no study has systematically compared both operative modalities against NSM within a unified analytical framework.

Given the heterogeneity in patient selection, fracture morphology, frailty burden, and outcome definitions across the literature, clinicians lack high-certainty, comparative evidence to guide treatment selection in this vulnerable population. The present systematic review and frequentist network meta-analysis therefore aims to integrate direct and indirect evidence across NSM, ADS, and PA to define the relative safety and effectiveness of each strategy. By leveraging network geometry and transitivity to synthesize disparate comparative datasets, we sought to establish a methodologically robust, clinically interpretable framework to support operative decision-making in contemporary geriatric type II odontoid fracture care.

## Methods

### Study design and reporting framework

This systematic review and frequentist network meta-analysis was performed in accordance with the Cochrane Collaboration Handbook for Systematic Reviews of Interventions [16] and reported following the Preferred Reporting Items for Systematic Reviews and Meta-Analysis – Network Meta-Analysis (PRISMA-NMA) extension (Supplementary Fig. 1) [17]. A protocol was registered a priori on the Prospective Register of Systematic Reviews (PROSPERO ID: CRD420251229550).

### Data sources and search strategy

A comprehensive search of PubMed/MEDLINE, Embase, and Cochrane Central Register of Controlled Trials (CENTRAL) was undertaken from inception to 20th October 2025 using predefined search terms (Supplementary Table 1). Reference and citation lists of included studies were manually examined to identify additional eligible articles. Titles, abstracts, and full texts were screened independently by two reviewers. Disagreements were resolved by consensus with a third reviewer. Inter-rater reliability was quantified using Cohen’s kappa (κ = 0.82), indicating near-perfect agreement.

### Eligibility criteria

Studies were eligible if they were: (i) randomized or observational comparative cohorts; (ii) directly compared at least two of NSM, ADS, or PA; (iii) included patients aged ≥ 60 years with Anderson-D’Alonzo type II odontoid fractures; (iv) reported at least one pre-defined primary outcome. We excluded studies that did not distinguish anterior from posterior surgical data, lacked extractable outcome data, or were non-comparative in design (editorials, letters, conference abstracts, case reports, or single-arm series). No exclusion criteria were applied on the basis of publication date or language. For duplicate or overlapping cohorts, the largest or most complete dataset was selected unless distinct outcomes were reported.

### Outcomes of interest

Primary outcomes were mortality, union (defined as trabecular bridging across the fracture or fusion site), stable non-union (defined as persistent non-bridging with minimal or no interfragmentary movement), unstable non-union (defined as persistent non-bridging with measurable displacement). Unstable non-union was considered clinically relevant when associated with persistent motion or displacement and was more likely to prompt secondary surgical intervention, whereas stable non-union often reflected a fibrous union without progressive instability.

Secondary outcomes included mechanical complications (defined as construct- or fixation-related adverse events, including malreduction, loss of reduction, implant malposition, loosening, or cut-out), systemic morbidity (defined as non-mechanical, medical complications, including infectious, pulmonary, cardiopulmonary, or thromboembolic events occurring during index admission), and secondary operation (defined as subsequent surgery for failed NSM, such as intolerance of immobilization or persistent pain, or revision surgery after initial operative fixation).

### Data extraction

Two reviewers independently extracted study characteristics, demographics, fracture morphology, treatment arm definitions, and event counts using a standardized form. Extracted interventions were operationalized as treatment strategies: NSM, ADS, and PA. Disagreements were resolved by consensus. When required, corresponding authors were contacted for clarification.

### Statistical analysis

All analyses were conducted in R v4.3.2 [42] using *netmeta* and *dmetar* packages [5]. Binary endpoints were synthesized using a frequentist random-effects Mantel–Haenszel network meta-analysis, generating pooled odds ratios (ORs) with 95% confidence intervals (CIs). Statistical significance was defined as *P* < 0.05 (two-tailed). A frequentist network meta-analysis framework was selected to maximize transparency and interpretability of effect estimates, particularly in anticipation of sparse events and predominantly observational data. This approach allows direct estimation of ORs and 95% CIs without reliance on prior distributions, facilitating clinical interpretation and alignment with established frequentist diagnostics for heterogeneity, inconsistency, and ranking. Bayesian approaches were considered but not pursued given the absence of robust priors and the potential for prior sensitivity to influence treatment rankings.

Inter-study heterogeneity was quantified using τ^2^ and I^2^ statistics via restricted maximum-likelihood estimation. Multi-arm studies were modelled using shared-comparator variance splitting to avoid double-counting. When adjusted comparative effect estimates were available, these were preferentially extracted for inclusion in the network meta-analysis, otherwise raw unadjusted arm-level event data was used. For sparse datasets or zero-event arms, a uniform continuity correction of 0.5 was applied [11].

Network geometry was visualized for each endpoint, and treatment ranking probabilities were estimated using P-scores (0–1 scale, with a higher score indicating better overall performance of the competing treatment), analogous to surface under the cumulative ranking curve [45]. Sensitivity analyses were performed for statistically significant outcomes by leave-one-out exclusion and excluding small study arms (*n* < 15) to assess robustness.

### Assessment of transitivity and network consistency

Transitivity was assessed qualitatively by evaluating comparability of study and participant characteristics across treatment contrasts. Global inconsistency was tested using the design-by-treatment interaction model, while local inconsistence was examined via node-splitting (*netsplit* function in *netmeta*) to compare direct and indirect estimates.

### Risk of bias and certainty of evidence

Risk of bias for non-randomized comparisons was assessed independently by two reviewers using Risk of Bias in Non-Randomized Studies of Interventions (ROBINS-I) [51], with consensus review for disagreements. Small-study effects and publication bias were examined using visual inspection of comparison-adjusted funnel plots and Egger’s regression test performed separately for each outcome [46]. Certainty of evidence for each treatment contrast was summarized using the Confidence in Network Meta-Analysis (CINeMA) framework [34], evaluating within-study bias, reporting bias, imprecision, heterogeneity, and incoherence.

## Results

**Fig. 1:**
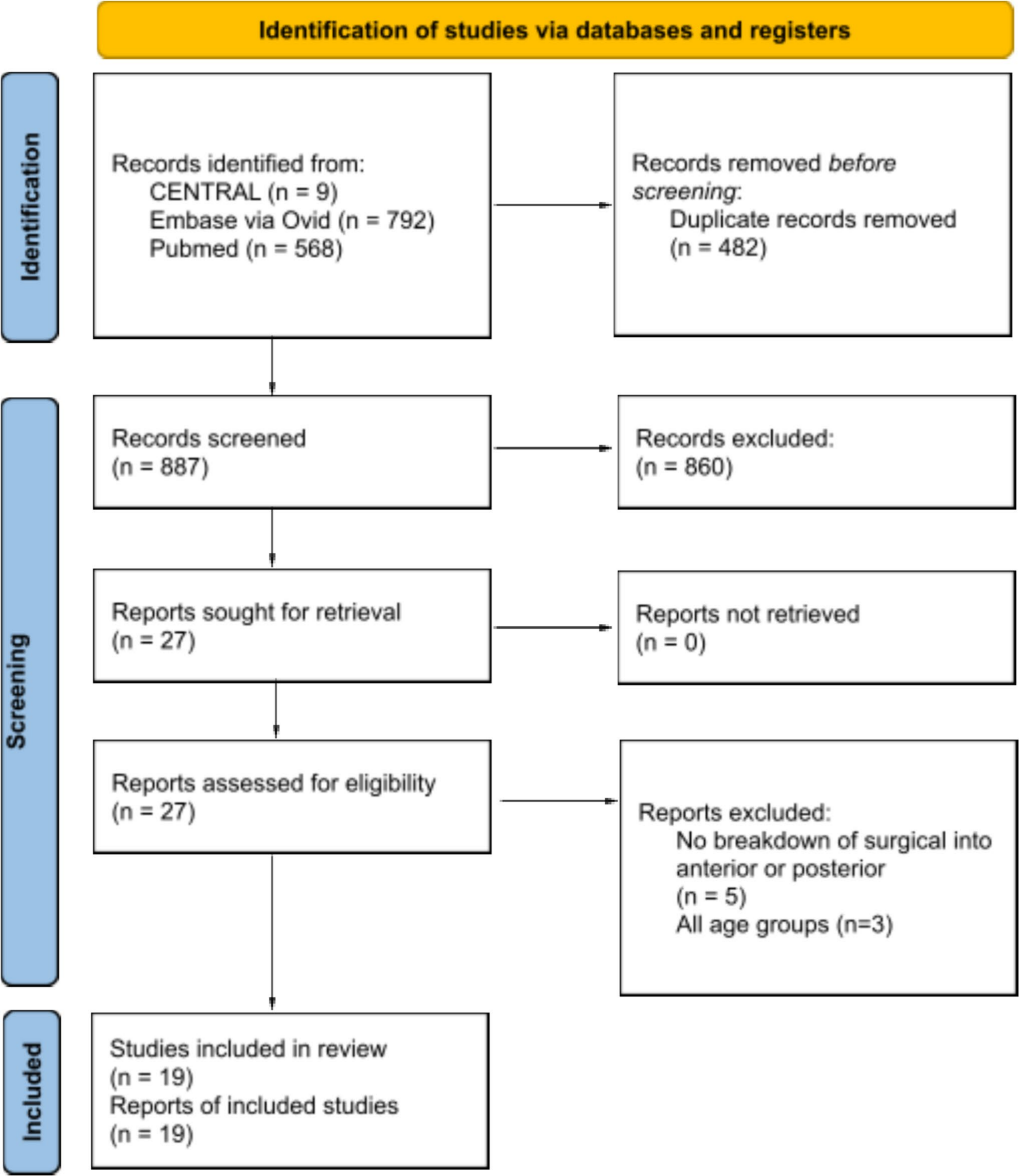
PRISMA Flow Diagram of Study Selection

Our search strategy identified 1369 records across major databases, of which 887 remained following removal of duplicates. After title and abstract screening, 27 full-text articles were reviewed, and 19 observational studies [1, 4, 7, 14, 15, 18, 23, 24, 27, 32, 33, 35, 36, 38, 39, 41, 43, 47, 49] met inclusion criteria, contributing a combined sample of 1242 elderly patients with Anderson-D’Alonzo type II odontoid fractures (Fig. 1). Of these, 475 patients (38.2%) underwent NSM, 340 (27.4%) underwent ADS, and 427 (34.4%) underwent PA. Baseline demographics and clinical characteristics were broadly comparable, though patients selected for PA exhibited a higher comorbidity burden (mean CCI 4.4 in PA versus 2.1 in NSM and 2.3 in ADS), and follow-up duration was longest in PA cohorts (mean 41.6 months) (though follow-up duration was not considered a baseline characteristic and did not influence treatment allocation or network transitivity). Complete study and operative characteristics are summarized in Tables 1 and 2.

**Table 1:** Baseline characteristics of included studies

Author (year)	Country, study design	Sample size (n)			Age (years) (mean ± SD or mean (range))			Sex (% male)			ASA grade (mean ± SD or mean (range))		
		NSM	ADS	PA	NSM	ADS	PA	NSM	ADS	PA	NSM	ADS	PA
Allia (2019)	France, RC	36	17	-	85.0 (70.0–105.0)	79.4 (70.0–90.0)	-	68.0		-	2.3 (1.0–4.0)	2.3 (1.0–3.0)	-
Joestl (2016)	Austria, RC	48	32	-	75.0 ± 6.0	71.0 ± 8.0	-	37.5	46.9	-	2.8 ± 0.7	2.6 ± 0.8	-
Chaudhary (2010)	Canada, RC	9	11	-	> 70*		-	33.3	54.5	-	NR	NR	-
Molinari (2013)	USA, RC	33	-	25	83.0 (65.0–91.0)	-	80.0*	48.5	-	60.0	NR	-	NR
Perry (2017)	USA, RC	94	-	17	83.0 (80.0–98.0)	-	84.0 (83.0–85.0)	31.4	-	54.2	NR	-	NR
Hamrick (2023)	USA, RC	35	-	24	80.6 ± 7.8	-	73.4 ± 7.6	31.4	-	54.2	NR	-	NR
Scheyerer (2013)	Switzerland, RC	14	17	16	80.1*	81.0*	82.4*	57.1	53.0	31.3	3.0 (2.0–5.0)	2.9 (2.0–4.0)	3.1 (2.0–4.0)
France (2012)	USA, RC	25	7	5	83.1 ± 6.5	81.0 ± 5.6		37.8			NR	NR	NR
Reinhold (2011)	USA, RC	4	3	18	83.0 (65.0–101.0)			80.0			NR	NR	NR
Kuntz (2000)	USA, RC	12	2	9	81.0*	73.5*	77.0*	57.0	100.0	67.0	NR	NR	NR
Jung (2023)	Germany, RC	19	35	18	83.2 ± 7.5			44.4			NR	NR	NR
Andersson (2000)	Sweden, RC	11	11	7	78.0 (66.0–99.0)			45.0	36.0	28.6	2.0 (1.0–3.0)	1.9 (1.0–3.0)	2.3 (2.0–3.0)
Moscolo (2021)	Italy, RC	11	21	2	73.5 (65.0–88.0)			76.5			NR	NR	NR
Huybregts (2024)	Netherlands, RC	124	29	74	76.6 ± 9.7	77.3 ± 9.1		40.0	50.0		NR	NR	NR
Platzer (2007)	Austria, RC	-	37	19	-	71.4*		-	44.6		-	2.2*	
Omeis (2009)	USA, RC	-	16	13	-	79.9 ± 1.6		-	38.0		-	NR	NR
Shousha (2019)	Germany, RC	-	47	86	-	74.2 ± 9.2	78.2 ± 9.2	-	40.0	30.0	-	NR	NR
Przkora (2006)	Germany, RC	-	7	1	-	80.5 (72.0–93.0)		-	43.0		-	3.0 (3.0–3.0)	3.0 (3.0–3.0)
Patterson (2017)	USA, RC	-	48	93	-	77.8 ± 6.5		-	41.5	46.5	-	NR	NR

Author (year)	CCI (mean ± SD or mean (range))			Type II Anderson–D’Alonzo fracture subtype (n)			Energy of injury (n)			Fracture displacement (mm) (mean ± SD or mean (range)) (direction)		
	NSM	ADS	PA	NSM	ADS	PA	NSM	ADS	PA	NSM	ADS	PA
Allia (2019)	NR	NR	-	NR	NR	-	Low (36)	Low (17)	-	2.5* (AP)		-
Joestl (2016)	NR	NR	-	NR	NR	-	Low (22); High (58)		-	4.8 ± 3.8 (AP)		-
Chaudhary (2010)	NR	NR	-	NR	NR	-	Low (16); High (4)		-	< 5.0* (NR)		-
Molinari (2013)	NR	-	NR	NR	-	NR	NR	-	NR	NR	-	NR
Perry (2017)	NR	-	NR	NR	-	NR	Low (9); High (4)	-	Low (5); High (1)	NR	-	NR
Hamrick (2023)	3.2 ± 2.3	-	3.2 ± 3.5	NR	-	NR	Low (35)	-	Low (19); High (5)	1.0 ± 2.1 (NR)	-	3.5 ± 3.2 (NR)
Scheyerer (2013)	NR	NR	NR	NR	NR	NR	Low (14)	Low (17)	Low (16)	1.9 (0.0–5.8) (LAT)		
France (2012)	1.1 ± 1.2	0.9 ± 0.9		NR	NR	NR	NR	NR	NR	NR	NR	NR
Reinhold (2011)	NR	NR	NR	NR	NR	NR	Low (69); High (8)			1.8 ± 2.3 (NR)	4.7 ± 3.8 (NR)	
Kuntz (2000)	NR	NR	NR	NR	NR	NR	Low (17); High (5)			4.5* (NR)	6.6* (NR)	
Jung (2023)	NR	NR	NR	A(3); B(16)	A(10); B(25)	A(1); B(17)	Low (68); High (4)			NR	NR	NR
Andersson (2000)	NR	NR	NR	NR	NR	NR	Low (6); High (5)	Low (9); High (2)	Low (4); High (3)	0.0 (0.0–0.0) (NR)	2.6 (0.0–8.0) (NR)	5.5 (3.0–8.0) (NR)
Moscolo (2021)	4.5 (4.0–5.0)	5.5 (3.0–13.0)	3.0*	A(2); B(9)	B(21)	C(2)	Low (20); High (14)			NR	NR	NR
Huybregts (2024)	NR	NR	NR	NR	NR	NR	NR	NR	NR	NR	NR	NR
Platzer (2007)	-	NR	NR	-	NR	NR	-	Low (19); High (37)		-	NR	NR
Omeis (2009)	-	NR	NR	-	NR	NR	-	Low (14); High (23)	Low (5); High (14)	-	6.3 ± 4.0 (NR)	
Shousha (2019)	-	NR	NR	-	B(47)	B(86)	-	NR	NR	-	NR	NR
Przkora (2006)	-	NR	NR	-	NR	NR	-	Low (7)	Low (1)	-	NR	NR
Patterson (2017)	-	NR	NR	-	NR	NR	-	NR	NR	-	NR	NR

Summary of demographic, clinical, and fracture-related characteristics for all comparative studies included in the meta-analysis. Columns report study author and publication year, country of study origin, study design, total sample size per surgical group, age distribution, sex distribution, American Society of Anesthesiologists (ASA) grade, Charlson Comorbidity Index (CCI), Anderson–D’Alonzo Type II odontoid fracture subtype (where reported), injury energy, and fracture displacement magnitude and direction. Data is presented as mean ± standard deviation, mean (range), or number (n). Where subgroup-specific data was unavailable, cells are marked as not reported (NR). Treatment groups include non-surgical management (NSM), anterior dens screw fixation (ADS), and posterior arthrodesis (PA)

Abbreviations & Symbols: ADS, anterior dens screw fixation; AP, anteroposterior, ASA, American Society of Anesthesiologists; CCI, Charlson Comorbidity Index; LAT, lateral; NR, not reported; NSM; non-surgical management; PA, posterior arthrodesis; RC, retrospective cohort. *Cell where mean and/or standard deviation and/or range not reported

**Table 2:** Operative, functional, and peri-treatment outcomes of included studies

Author (year)	Technique description (n)			Operative time (mins) (mean ± SD or mean (range))			VAS score change (mean ± SD or mean (range))			NDI score change (mean ± SD or mean (range))			Length of stay (days) (mean ± SD or mean (range))			Follow-up (months) (mean ± SD or mean (range))			Postoperative functional activity and/or discharge disposition (n)		
	NSM	ADS	PA	NSM	ADS	PA	NSM	ADS	PA	NSM	ADS	PA	NSM	ADS	PA	NSM	ADS	PA	NSM	ADS	PA
Allia (2019)	Rigid collar (36)	Anterior screw (Böhler technique) (17)	-	-	NR	-	NR	NR	-	NR	NR	-	8.1*		-	12.0*		-	NR	NR	-
Joestl (2016)	Halo vest (48)	Anterior screw (Böhler technique) (32)	-	-	140.0 (90.0–210.0)	-	NR	NR	-	NR	NR	-	NR	NR	-	24.0*		-	No functional impairment (34); partial (7); complete (3)	No functional impairment (25); partial (4)	-
Chaudhary (2010)	Rigid collar (9)	Anterior odontoid screw (11)	-	-	NR	-	0.7*	1.2*	-	NR	NR	-	NR	NR	-	9.0*	4.0*	-	Partial functional impairment (2)	Partial functional impairment (1)	-
Molinari (2013)	Rigid collar (33)	-	Posterior screw with instrumented fusion (25)	-	-	109.0 (56.0–229.0)	NR	-	NR	NR	-	NR	NR	-	NR	13.9 (2.0–48.0)	-	13.7 (0.3–48.0)	NR	-	NR
Perry (2017)	Rigid collar (94)	-	Posterior screw with instrumented fusion (17)	-	-	NR	1.3 (0.0–4.0)	-	1.8 (0.0–7.0)	13.0 (0.0–28.0)	-	18.1 (0.0–48.0)	NR	-	NR	52.0 ± 9.0	-	79.0 ± 11.5	Home discharge (83); other location (11)	-	Home discharge (12); other location (5)
Hamrick (2023)	Rigid collar (35)	-	Posterior screw with instrumented fusion (24)	-	-	NR	2.3 ± 2.8	-	2.3 ± 2.8	43.4 ± 15.7	-	46.2 ± 21.6	2.8 ± 3.8	-	4.7 ± 3.7	NR	-	NR	Home discharge (18); rehabilitation center (1); skilled nursing facility (2); assisted-living (9); hospice (4)	-	Home discharge (13); rehabilitation center (1); skilled nursing facility (4); assisted-living (4); hospice (1)
Scheyerer (2013)	Rigid collar (14)	Anterior screw (16); Traction screw (partially threaded) (1)	Posterior screw (Harms technique) (16)	-	NR	NR	NR	NR	NR	NR	NR	NR	NR	NR	NR	31.1 ± 19.0			NR	NR	NR
France (2012)	Cervical collar (9); Halo vest (16)	Anterior screw (7)	Posterior screw with instrumented fusion (5)	-	NR	NR	NR	NR	NR	NR	NR	NR	NR	NR	NR	34.1 ± 37.3			NR	NR	NR
Reinhold (2011)	Rigid collar (52); Soft collar (6); Halo vest (5)	Anterior odontoid screw (6)	Posterior screw with instrumented fusion (17); Transarticular screw (8)	-	NR	NR	NR	NR	NR	NR	NR	NR	9.9 ± 8.0	5.7 ± 4.0		6.0*	5.7*		NR	NR	NR
Kuntz (2000)	Halo vest (8); Minnerva brace (2); Miami J collar (2)	Anterior odontoid screw (2)	Transarticular screw with instrumented fusion (modified Gallie technique) (9)	-	NR	NR	NR	NR	NR	NR	NR	NR	13.0*	14.0*		13.0*	17.0*		NR	No functional impairment (2)	No functional impairment (6)
Jung (2023)	Rigid collar (12); soft collar (7)	Anterior odontoid screw (35)	Posterior screw with instrumented fusion (18)	-	51.5 ± 32.1	169.1 ± 58.7	NR	NR	NR	NR	NR	NR	9.4 ± 7.7	12.0 ± 4.3	22.5 ± 17.2	2.7 ± 2.1			NR	NR	NR
Andersson (2000)	Rigid collar (11)	Anterior screw (Böhler technique) (11)	Posterior screw with instrumented fusion (7)	-	NR	NR	NR	NR	NR	NR	NR	NR	NR	NR	NR	51.0 (24.0–89.0)			NR	NR	NR
Moscolo (2021)	Rigid collar (9); Halo vest (2)	Anterior screw (21)	Posterior screw (Harms technique) (2)	-	NR	NR	6.0*	3.0 (0.0–5.0)	6.5 (6.0–7.0)	42.7 (30.0–55.5)	20.0 (0.0–37.5)	37.5 (32.5–42.5)	NR	NR	NR	(3.0–6.0)*			NR	NR	NR
Huybregts (2024)	Rigid collar (96); Halo vest (28)	Anterior odontoid screw (29)	Posterior screw with instrumented fusion (74)	-	NR	NR	25.0 ± 5.7	28.0 ± 7.3		15.0 ± 1.7	16.0 ± 2.4		NR	NR	NR	13.0*			NR	NR	NR
Platzer (2007)	-	Double small-fragment screw (cannulated) (37)	Posterior wiring with bone graft (modified Brooks & Jenkins technique) (19)	-	NR	NR	-	NR	NR	-	NR	NR	-	NR	NR	-	(12.0–24.0)*		-	NR	NR
Omeis (2009)	-	Anterior odontoid screw (16)	Lateral mass screw (9); Transarticular screw with instrumented fusion (modified Gallie technique) (13)	-	140.0 (70.0–340.0)		-	NR	NR	-	NR	NR	-	14.0 ± 8.6		-	9.0 ± 3.2		-	Home discharge (23); skilled nursing facility (2); home to skilled nursing facility (3); perioperative death (1)	
Shousha (2019)	-	Double screw (47)	Transarticular screw with bone graft (86)	-	64.5 ± 25.6	116.6 ± 47.0	-	2.4 ± 1.8	2.9 ± 2.1	-	NR	NR	-	17.4 ± 15.0	30.0 ± 25.0	-	29.3 ± 10.4	32.0 ± 14.6	-	NR	NR
Przkora (2006)	-	Double screw (7)	Posterior screw with instrumented fusion (1)	-	NR	NR	-	NR	NR	-	NR	NR	-	31.4 (16.0–64.0)		-	18.0*		-	NR	NR
Patterson (2017)	-	Anterior odontoid screw (93)	Posterior screw (93)	-	86.0 (74.0–99.0)	158.0 (145.0–171.0)	-	NR	NR	-	NR	NR	-	6.3 (4.3–8.4)	5.3 (3.7–7.1)	-	NR	NR	-	Other (non-home) discharge location (14)	Other discharge (non-home) location (34)

Summary of operative characteristics, patient-reported outcomes, length of hospital stay, follow-up duration, and postoperative functional activity or discharge disposition for all comparative studies included in the meta-analysis. Columns report surgical technique by treatment group, operative time, change in visual analogue scale (VAS) pain score, change in Neck Disability Index (NDI) score, length of hospital stay, duration of follow-up, and reported postoperative functional activity or discharge destination. Data is presented as mean ± standard deviation, mean (range), or number (n). Where subgroup-specific data was unavailable, cells are marked as not reported (NR). Treatment groups include non-surgical management (NSM), anterior dens screw fixation (ADS), and posterior arthrodesis (PA)

Abbreviations & Symbols: ADS, anterior dens screw fixation; AP, anteroposterior, ASA, American Society of Anesthesiologists; CCI, Charlson Comorbidity Index; NDI, Neck Disability Index; NR, not reported; NSM; non-surgical management; PA, posterior arthrodesis; VAS, Visual Analogue Scale. *Cell where mean and/or standard deviation and/or range not reported

Across all primary endpoints, a complete three-node treatment network was formed (Figs. 3A, 4A, 5A, and 6A), with abundant direct evidence between NSM and each surgical modality, and a mixture of direct and indirect contrasts informing ADS-PA comparisons (Figs. 3C, 4C, 5C, and 6A). Network geometry was well balanced, with no evidence of disconnected subgraphs or isolated interventions.

### Mortality

**Fig. 2:**
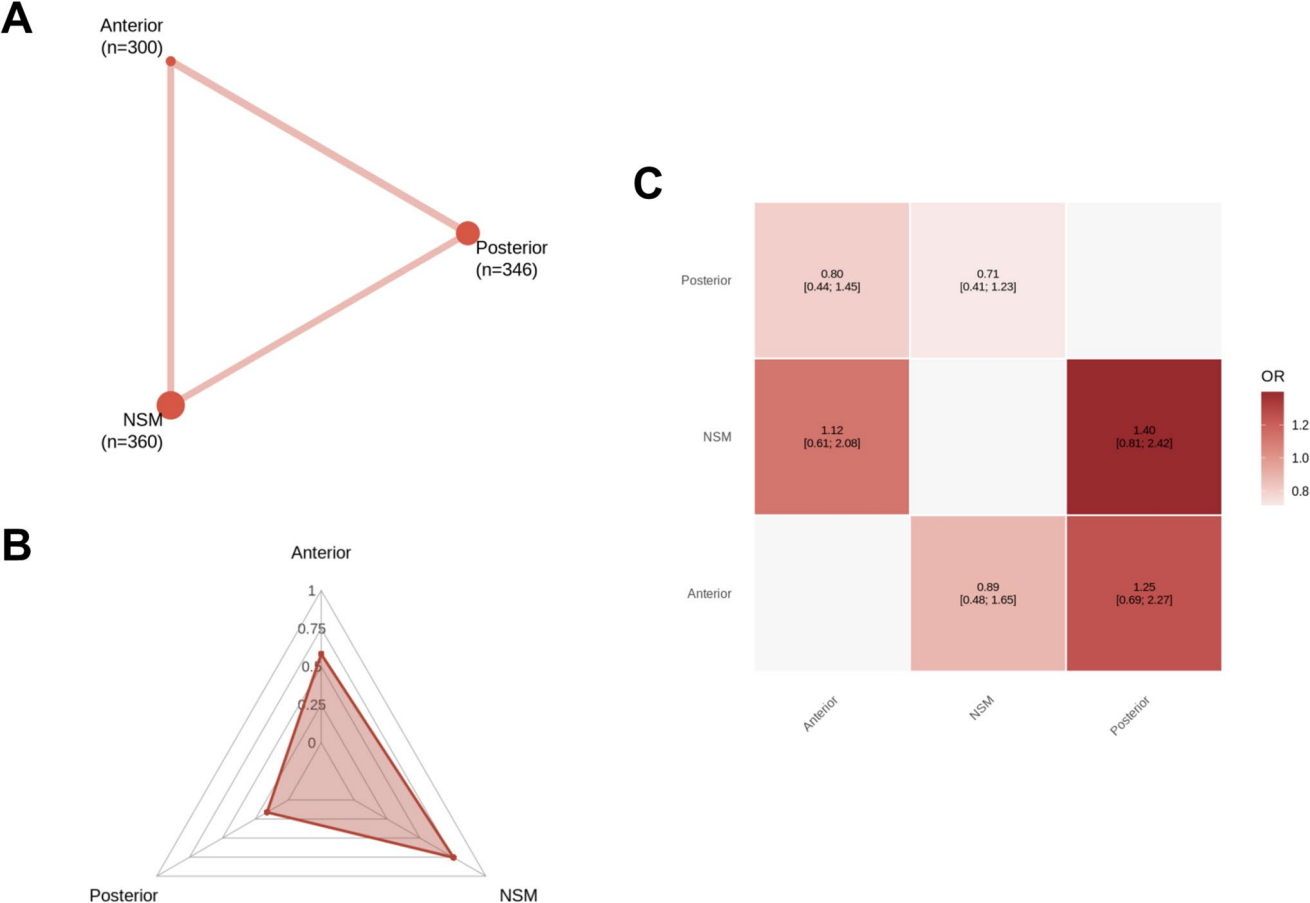
Network meta-analysis for mortality. **A** Network plot showing direct comparisons between interventions. **B** P-scores radar plot **C** League table heatmap of intervention effects. Abbreviations: Anterior = Anterior Dens Screw Fixation. NSM = Non-Surgical Management. Posterior = Posterior Arthrodesis

Mortality was reported in seventeen studies [1, 7, 14, 15, 23, 24, 27, 32, 33, 35, 36, 38, 39, 41, 43, 47, 49] (*n* = 1006), with 191 deaths recorded (18.9%). No statistically significant differences were detected between NSM, ADS, and PA (Fig. 2C). Mortality was reported at heterogeneous timepoints across studies (including in-hospital, 30-day, or longest available follow-up), precluding stratified timepoint-specific analyses. Accordingly, results reflect overall mortality risk rather than early versus late survival effects.

### Fusion and non-union outcomes

**Fig. 3:**
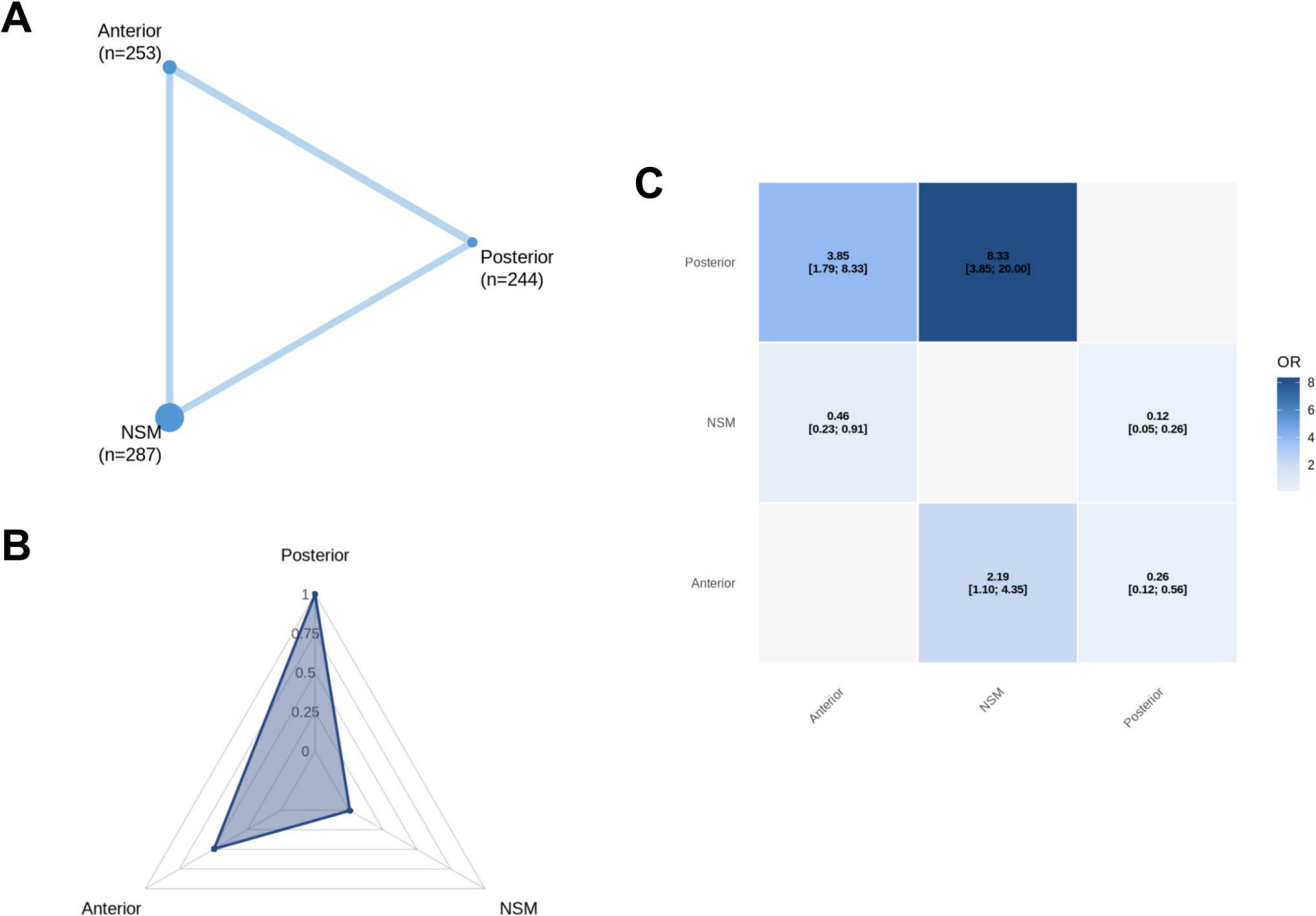
Network meta-analysis for union. **A** Network plot showing direct comparisons between interventions. **B** P-scores radar plot **C** League table heatmap of intervention effects. Abbreviations: Anterior = Anterior Dens Screw Fixation. NSM = Non-Surgical Management. Posterior = Posterior Arthrodesis

Union was reported sixteen studies [1, 4, 7, 18, 23, 24, 27, 32, 33, 35, 38, 39, 41, 43, 47, 49] (*n* = 784), with 539 events recorded (68.8%). Compared with NSM, ADS significantly increased the odds of union (OR: 2.19; 95% CI: 1.10–4.35), while PA conferred an even greater improvement relative to NSM (OR: 8.35; 95% CI: 3.79–18.4) and ADS (OR: 0.26; 95% CI: 0.12–0.56). These findings are illustrated in the union league table (Fig. 3C) and remained stable across leave-one-out and small-arm exclusions.

**Fig. 4:**
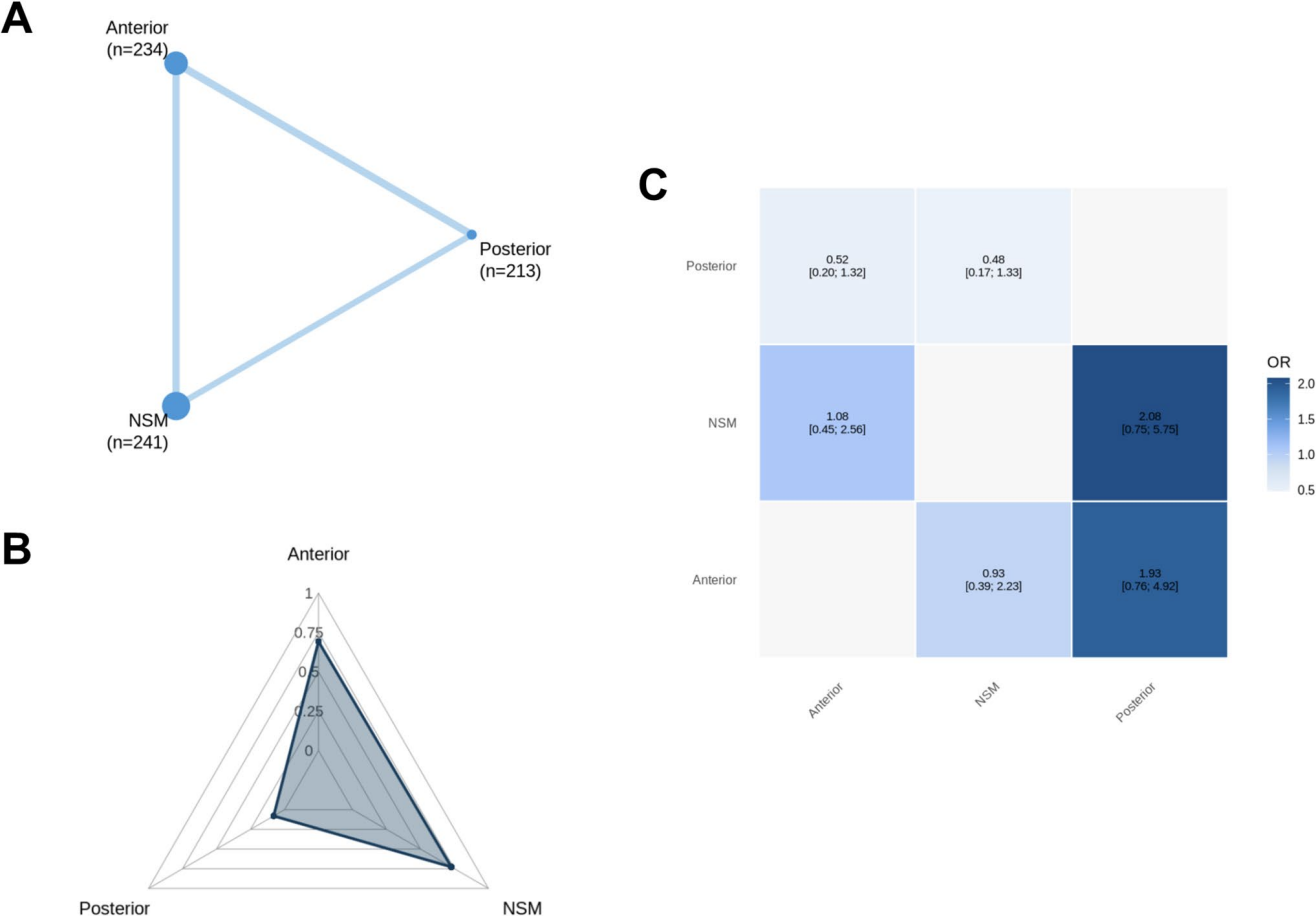
Network meta-analysis for stable non-union. **A** Network plot showing direct comparisons between interventions. **B** P-scores radar plot **C** League table heatmap of intervention effects. Abbreviations: Anterior = Anterior Dens Screw Fixation. NSM = Non-Surgical Management. Posterior = Posterior Arthrodesis

Stable non-union was reported in thirteen studies [1, 4, 7, 18, 23, 27, 32, 33, 35, 39, 41, 47, 49] (*n* = 688), with 131 events recorded (19.0%) These were dispersed evenly among NSM, ADS, and PA, and no statistically significant differences emerged between any treatment contrasts, with all confidence intervals crossing unity (Fig. 4C).

**Fig. 5:**
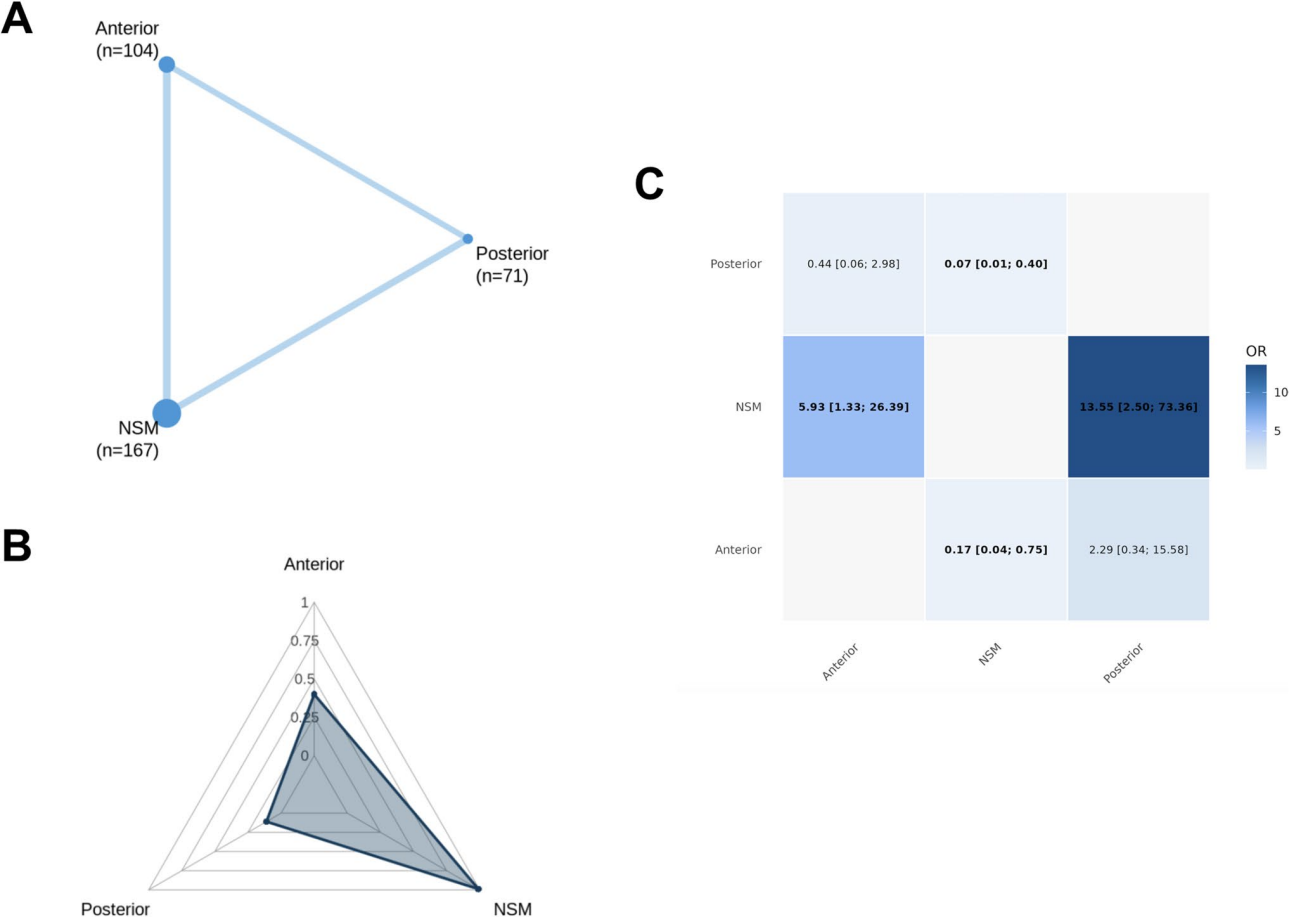
Network meta-analysis for unstable non-union. **A** Network plot showing direct comparisons between interventions. **B** P-scores radar plot **C** League table heatmap of intervention effects. Abbreviations: Anterior = Anterior Dens Screw Fixation. NSM = Non-Surgical Management. Posterior = Posterior Arthrodesis

Unstable non-union was reported in eight studies [1, 4, 7, 15, 23, 32, 33, 35] (*n* = 342), with 53 events recorded (15.5%). NSM was associated with significantly higher odds of unstable non-union compared with both operative strategies. ADS reduced the odds of unstable non-union relative to NSM (OR: 5.93; 95% CI: 1.33–26.35), and PA produced an even greater reduction compared with NSM (OR: 13.55; 95% CI: 2.50–73.36). No statistically significant difference was detected between ADS and PA (Fig. 5C).

### Complications and secondary operations

Mechanical complications were reported in fourteen studies [1, 4, 23, 24, 27, 32, 33, 35, 38, 39, 41, 43, 47, 49] (*n* = 727), with 59 events recorded (8.1%). Event counts were low across all strategies, and no statistically significant differences were demonstrated between NSM, ADS, and PA (Supplementary Fig. 1 C).

Systemic morbidity was reported in thirteen studies [1, 4, 23, 24, 27, 32, 33, 35, 36, 38, 39, 41, 49] (*n* = 796), with 174 events recorded (21.9%). ADS was associated with significantly more odds of systemic morbidity than NSM (OR: 1.87; 95% CI: 1.02–3.44), as was PA (OR: 2.29; 95% CI: 1.20–4.36). No significant difference was observed between ADS and PA (Supplementary Fig. 2 C).

Secondary operations were reported in thirteen studies [4, 7, 15, 23, 27, 33, 35, 36, 38, 39, 43, 47, 49] (n = 875), with 89 events recorded (10.2%). No treatment contrast reached statistical significance (Supplementary Fig. 3 C).

### Treatment hierarchy, network coherence, and heterogeneity

Ranking probabilities (P-scores) reflected the direction and magnitude of effect estimates. PA consistently ranked highest for union (Fig. 3B) and lowest for unstable non-union (Fig. 5B), while NSM ranked most favorably for mortality (Fig. 2B), stable non-union (Fig. 4B), and systemic morbidity (Supplementary Fig. 3 C). ADS typically occupied an intermediate position. Rankings for sparse outcomes, such as unstable non-union and mechanical complications, demonstrated greater uncertainty (Supplementary Fig. 4). To aid clinical interpretation, absolute event proportions pooled by treatment strategy in the included studies are summarized in Supplementary Table 4.

Network consistency was supported by all statistical diagnostics. There was no evidence of global inconsistency using design-by-treatment interaction modelling across outcomes: mortality (*P* = 0.39), union (*P* = 0.06), stable non-union (*P* = 0.06), unstable non-union (*P* = 0.12), mechanical complications (*P* = 0.93), systemic morbidity (*P* = 0.14). Node-splitting analyses demonstrated agreement between direct and indirect estimates for every treatment contrast (Supplementary Fig. 5 & 6). Heterogeneity was low to moderate depending on the endpoint: union (I^2^ = 42.5%), stable non-union (I^2^ = 12.6%), unstable non-union (I^2^ = 40.3%), systemic morbidity (I^2^ = 24.7%), and negligible for mortality (I^2^ = 0.0%) and mechanical complications (I^2^ = 0.0%).

Funnel plots showed symmetry, with all studies contained within the 95% CI pyramidal lines (Supplementary Fig. 7). Egger’s regression indicated no small-arm effects (*P* > 0.05 for all measured outcomes). ROBINS-I assessments identified predominantly low-to-moderate risk of bias, with confounding the most frequent concern (Supplementary Table 2). CINeMA grading indicated high certainty for mortality, with minimal downgrading due to high imprecision, within-study bias, and heterogeneity (Supplementary Fig. 8).

## Discussion

Across nineteen observational studies comprising 1242 geriatric (≥ 60 years) patients with type II odontoid fractures, this network meta-analysis provides a comparative evaluation of NSM, ADS, and PA, contextualized with unique physiological vulnerabilities of the ageing cervical spine. Our principal findings demonstrate no mortality differences between strategies, while fusion outcomes consistently favored surgical intervention (most notably PA), and unstable non-union was significantly more common following NSM than either operative approach. Mechanical complication and secondary operation were broadly comparable, whereas systemic morbidity was more frequent after surgery. Together, this data reinforces a pragmatic, morphology- and frailty-informed framework in which PA offers the most reliable structural correction, ADS provides motion-preserving fixation in selected anatomies, and NSM remains appropriate for physiologically fragile patients whose survival trajectory is dominated by comorbidity rather than fixation strategy.

### Clinical interpretation and patient selection

When translated into clinical decision-making, these findings should be interpreted as probabilistic guidance rather than prescriptive rules. PA is most likely to benefit patients with displaced, unstable, or irreducible fractures who can tolerate surgery and in whom achieving structural stability is a priority. ADS fixation may be appropriate for selected patients with reducible fracture morphology, preserved bone quality, and a desire to maintain atlantoaxial motion. NSM remains a reasonable option for very frail patients, those with limited physiological reserve, or those unable to tolerate surgery, recognizing their higher risk of non-union but similar overall mortality. Importantly, these recommendations are derived from observational data and are subject to confounding by indication. They should therefore be individualized through multidisciplinary discussion that incorporates patient goals, frailty, comorbidity burden, and tolerance of immobilization rather than interpreted as evidence of causal superiority.

### Reframing success: between structural healing and physiological reserve

These findings foreground a central tension in geriatric odontoid fracture care: success is multidimensional. Radiographic union, mechanical alignment, neurological preservation, pain control, functional recovery, and survival each contribute differently depending on patient context [54]. Although surgery clearly improves fusion, with PA achieving over eightfold higher odds of union compared with NSM, and reduces unstable non-union, structural gains must be balanced against the physiological cost of operative intervention in older adults [12, 38]. Several cohort studies have demonstrated that early post-operative morbidity, particularly dysphagia, pulmonary complications, and dependence on enteral feeding, may attenuate the theoretical survival benefit of fixation [2, 6, 10, 13, 48]. This pattern is echoed in the results of our analysis with greater odds of systemic morbidity after ADS and PA despite similar survival across all groups.

Stable and unstable non-union represent distinct clinical entities. Stable non-union often reflects a fibrous union with minimal motion, which may remain clinically tolerable in older adults without neurological compromise [54]. In contrast, unstable non-union is more likely to manifest with persistent pain, progressive displacement, or functional instability, and frequently precipitates secondary surgical intervention. In the present analysis, non-union outcomes were defined as radiographically as reported by the primary studies, and clinical symptom data were inconsistently reported. All analyses were performed on an intent-to-treat basis according to initial management strategy, with secondary operations analyzed as a separate endpoint rather than reclassifying union status.

Age-related physiological shifts further complicate interpretation. Sarcopenia, impaired cough reflex, reduced chest wall compliance, diminished laryngeal sensitivity, endothelial dysfunction, and blunted baroreflexes combine to heighten the perioperative risk associated with prone surgical positioning, airway manipulation, and general anesthesia [31]. These mechanisms help reconcile why NSM, though structurally inferior, yields comparable mortality, where survival in this cohort reflects biological rather than surgical selection. Thus, treatment selection must privilege individualized physiological reserve, not merely chronological age.

Mortality therefore warrants specific contextualization in this population. Mortality in geriatric odontoid fracture is driven predominantly by baseline physiological reserve, comorbidity burden, and injury-related factors rather than fixation strategy alone [48]. Early mortality is often attributable to acute medical complications, aspiration, or deconditioning, whereas later mortality reflects frailty, institutionalization, and competing systemic disease. Given heterogeneous reporting of mortality timepoints across studies, the present analysis should be interpreted as comparing overall mortality risk rather than early versus late survival effects. Accordingly, the absence of a statistically significant mortality difference between treatment strategies should not be construed as equivalence. These observations must also be interpreted in the context of confounding by indication, whereby treatment allocation is strongly influenced by baseline frailty, comorbidity, and injury characteristics that independently affect mortality and morbidity.

### Biomechanical and biological foundations of the observed treatment hierarchy

The consistent superiority of PA for union likely reflects its biomechanical advantage in restoring rigid multiplanar stability across the C1-C2 complex. Posterior constructs (such as Goel-Harms C1 lateral mass-C2 pars/pedicle fixation or Magerl trans-articular screws) neutralize flexion–extension and rotational shear while reconstituting the posterior tension band, thereby promoting an osteogenic microenvironment [29]. In older adults with osteopenic or osteoporotic bone, this rigid biomechanical constraint is crucial for preventing micromotion across the fracture plane that would otherwise impede chondral ossification and trabecular bridging. However, osteoporosis was infrequently formally quantified across included studies, with few reporting objective bone density measures such as DEXA-derived T-scores. Consequently, associations between bone quality and treatment failure or non-union were largely inferred rather than empirically tested, and the present discussion reflects biomechanical plausibility rather than causal attribution [12]. Multiple biomechanical studies in cadaveric odontoid fracture models corroborate this, where posterior fixation reduces motion to < 1–2 degrees in flexion and rotation, while ADS constructs permit greater translational shear under physiologic load [50]. These advantages position PA as the most biomechanically robust strategy for displaced, irreducible, comminuted, or translational injuries, and for cases where osteoporosis undermines anterior screw purchase.

Conversely, ADS directly compresses the fracture line and preserves C1-C2 rotation, offering a targeted, motion-preserving strategy in anatomy that permits a favorable screw trajectory. ADS retains a central role for patients with reducible fractures, intact transverse ligament integrity, and favorable odontoid lines, where its biomechanical appeal is further strengthened by shorter operative duration, supine surgical positioning, and avoidance of the physiological demands of prone surgery [30]. However, its success is acutely dependent on fracture reducibility, bone stock, transverse ligament integrity, and odontoid morphology (such as Grauer subtype IIB versus IIC). ADS is also vulnerable to osteoporosis, where diminished screw purchase and altered trabecular architecture reduce construct reliability. These constraints help explain the intermediate performance of ADS in our network, where fusion outcomes surpassed NSM but remained inferior to PA.

NSM, by contrast, relies entirely on external immobilization. Rigid collars cannot neutralize internal shear or micromotion across the C1-C2 articulation [44]. Osteoporotic bone, diminished osteoblastic potential, and age-related impairment of angiogenic signaling (reduced VEGF and TGF-β responsiveness) produce a biological milieu ill-suited to trabecular bridging [60]. This mechanistic triad likely explains why unstable non-union was significantly more frequent after NSM (OR 5.93 versus ADS; OR 13.55 versus PA). Nonetheless, many stable fibrous unions remain clinically silent, allowing NSM to remain viable for select patients prioritizing comfort or avoidance of perioperative risk.

Although collar-based treatment is often considered the least physiologically demanding option, its effectiveness in routine practice may be constrained by real-world adherence [20, 52]. Halo vest immobilization carries device-specific hazards, including pin tract infection, loosening, dysphagia, pressure injury, and impaired horizontal gaze, making it poorly tolerated in frail adults and generally unsuitable as first-line immobilization. In the present analysis, halo vests and rigid cervical collars were therefore grouped under NSM, as most comparative studies did not report outcomes separately by immobilization modality and contemporary practice has largely abandoned halo use in older adults. Rigid cervical collars are therefore the preferred NSM modality in contemporary practice, but their efficacy is wholly dependent on continuous wear for 10–12 weeks [9, 19]. Compliance in older adults is frequently limited by discomfort, cognitive impairment, skin breakdown, and carer support. Consequently, the structural shortcomings of NSM must be interpreted through the lens of adherence rather than protocol fidelity, recognizing that collar-based care, when well tolerated, has not been consistently associated with neurological deterioration.

### Integrating fracture morphology, frailty, and goals of care into treatment selection

Morphological factors must guide treatment decisions. Displaced, irreducible, or comminuted injuries, particularly those exceeding 4–5 mm translation or demonstrating atlantoaxial instability, are poorly suited to NSM or ADS and logically favor PA. Conversely, minimally displaced fractures or those presenting in patients with dementia, severe cardiopulmonary disease, or limited life expectancy may be better served by NSM, particularly when functional goals emphasize pain control and avoidance of intervention. Frailty indices, sarcopenia markers, pre-operative swallow assessment, and pulmonary function provide more reliable predictors of post-operative complications than chronological age alone. These considerations align with contemporary paradigms in geriatric trauma, where biologic age and phenotypic vulnerability increasingly replace conventional chronological thresholds.

**Fig. 6:**
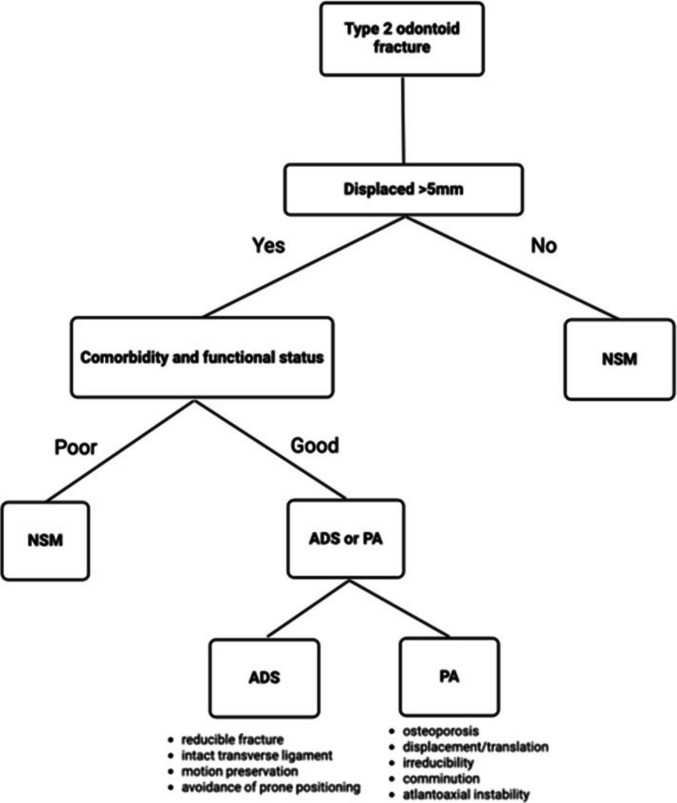
Pragmatic morphology-frailty-mechanics decision algorithm for the management of type II odontoid fractures in geriatric populations. This flowchart outlines a structured approach to treatment selection incorporating fracture displacement (> 5 mm), physiological reserve, comorbidity burden, and anatomical suitability for fixation. Minimally displaced fractures or patients with poor physiological reserve favour non-surgical management (NSM). Among fit patients with displaced fractures, either anterior dens screw (ADS) or posterior arthrodesis (PA) may be selected based on fracture reducibility, transverse ligament integrity, and biomechanical stability requirements. ADS is prioritised for reducible fractures with favourable morphology and when motion preservation or avoidance of prone surgical positioning is desirable, whereas PA is preferred for irreducible, comminuted, osteoporotic, or overtly unstable C1-C2 injuries. This algorithm synthesises clinical, biomechanical, and geriatric considerations to guide personalised treatment selection. Abbreviations: Anterior = Anterior Dens Screw Fixation. NSM = Non-Surgical Management. Posterior = Posterior Arthrodesis

Patient selection is therefore the fulcrum upon which all treatment decisions rest, and chronological age is an inadequate discriminator. Functional age, frailty phenotype, comorbidity burden, bone quality, and fracture morphology together determine the physiological capacity to withstand operative stress. NSM remains appropriate for minimally displaced fractures, acceptable alignment, absence of myelopathy, and patients with high comorbidity burden or cognitive impairment who prioritize avoidance of surgery. ADS is best suited to reducible fractures with favorable orientation, intact transverse ligament support, and patients in whom motion preservation or avoidance of prone surgical positioning is desirable. PA is preferred for displaced, irreducible, or unstable fractures, or where osteoporotic bone compromises anterior fixation. The challenge is to reconcile the structural advantages of surgery with its perioperative risks in older adults, matching treatment to individual physiology and care goals. Figure 6 provides a pragmatic morphology-frailty-mechanics framework synthesizing these decision variables into a clinically actionable algorithm.

From a clinical implementation perspective, the present framework is best applied within a multidisciplinary setting that integrates spinal surgery, geriatrics, anesthesiology, rehabilitation medicine, and allied health services. In older adults, outcomes following odontoid fracture are strongly influenced by post-treatment care, including early mobilization, swallow assessment, pulmonary hygiene, and structured rehabilitation. Operative strategies may require proactive dysphagia screening and respiratory support, whereas NSM depends heavily on adherence to immobilization, skin care, and carer support. Fracture management should therefore extend beyond fixation strategy alone and incorporate peri-treatment rehabilitation capacity, discharge planning, and patient-centered goals of care.

### Contextualizing our results within the existing evidence base

Our findings align with prior meta-analyses showing higher odds of fusion with PA [21, 53] and confirm the well-recognized dysphagia burden associated with the anterior approach [10]. They also corroborate earlier cohort studies in which mortality differences between operative and non-operative groups diminished after adjusting for comorbidity and functional status [37, 58]. Importantly, our demonstration of elevated systemic morbidity after surgery mirrors broader evidence in hip fracture, thoracolumbar trauma, and cervical spondylotic myelopathy populations, where frailty rather than surgical technique drives 90-day mortality and readmission [25, 26, 56].

A key limitation of the current evidence landscape is the near-absence of functional outcomes [54]. Measures such as the Neck Disability Index (NDI), Short Form 36 Health Survey (SF-36) physical and mental component scores, EuroQol 5 (EQ-5D), or targeted geriatric functional scales were rarely reported across included studies, despite their centrality to outcome appraisal in older adults. Radiographic union does not always translate into improved independence, swallow safety, or quality of life, and a stable fibrous union may offer acceptable pain control and functional preservation in selected patients [57]. Similarly, inconsistent reporting of dysphagia metrics limits interpretation of morbidity differences between ADS and PA. Future prospective studies incorporating patient-reported outcome metrics (PROMs) and validated functional scales are needed to define what constitutes meaningful recovery in this population.

### Methodological considerations

This study is limited by the observational nature of all included evidence, resulting in susceptibility to confounding by indication. In geriatric odontoid fracture care, treatment selection is closely linked to baseline frailty, comorbidity burden, fracture displacement, reducibility, and tolerance of immobilization, each of which independently influences mortality and morbidity. As a result, comparisons (particularly for survival and systemic complications) may reflect differences in patient selection rather than causal effects of treatment strategy, despite network transitivity being satisfied and no evidence of global or local inconsistency.

Outcome definitions for fracture healing varied across included studies, with differences in how union, stable (fibrous) union, and non-union were defined and assessed. Imaging modalities and follow-up schedules were heterogeneous, ranging from plain radiography to computed tomography at variable timepoints, which may have influenced classification of healing states. Outcomes were therefore extracted and synthesized according to the definitions used in the primary studies, and distinctions between osseous union and clinically stable fibrous union should be interpreted with caution when comparing pooled estimates across cohorts. In addition, heterogeneity in fracture classification may have influenced network transitivity; although most cohorts focused on Anderson-D’Alonzo type II fractures, reporting of Grauer subtypes was inconsistent, and differences in reducibility, comminution, and fracture orientation may have contributed to treatment selection and outcome variability.

Event counts for several outcomes, including unstable non-union, mechanical complications, and systemic morbidity, were sparse, widening CIs and reducing the precision of treatment rankings. Although global and local inconsistency tests revealed no significant incoherence, some contrasts (such as ADS versus PA) relied partially on indirect evidence. Frailty and comorbidity indices (such as the CCI or American Society of Anesthesiologists (ASA) physical status grade) were variably reported, precluding consistent adjustments for physiological reserve across studies. Sensitivity analyses restricted to studies with comparable baseline frailty, comorbidity burden, or fracture morphology were therefore not feasible, as these characteristics were inconsistently reported and variably defined. Similarly, age-stratified effect estimates could not be reliably derived from aggregate data, as most studies reported only overall cohort means or medians without stratified outcomes. In addition, functional outcomes, pain metrics, swallowing or aspiration risk, discharge destination, and quality-of-life measures were inconsistently reported, limiting the ability to contextualize structural outcomes with patient-centered endpoints. Most outcomes were reported as binary endpoints at fixed follow-up intervals. Future prospective studies would benefit from time-to-event analyses for mortality, reoperation, and complication burden. Addressing age-, frailty-, and morphology-specific treatment effects will likely require prospective cohorts or individual patient-level meta-analysis with standardized reporting.

### Future directions

Future work should prioritize prospective, multicenter comparative cohorts that stratify by fracture morphology, bone quality, frailty indices, and dysphagia risk. Standardized definitions of union, stable non-union, and systemic complications (including swallow evaluations, pulmonary metrics, and validated geriatric functional scales) would improve comparability. Biomechanical modelling and finite element studies could refine thresholds for displacement and fracture line orientation predictive of success in ADS versus PA. Finally, incorporating patient-reported outcomes, deglutition assessments, and global health metrics would better define what “successful treatment” means for this uniquely vulnerable population.

## Conclusion

In geriatric patients with type II odontoid fractures, mortality does not differ significantly across NSM, ADS, and PA, reflecting the dominant influence of comorbidity and physiological reserve, Surgery, particularly PA, provides the most reliable fusion and lowest unstable non-union risk, while NSM reduces systemic morbidity at the cost of higher structural failure. Treatment should therefore be individualized, integrating fracture morphology, frailty, bone quality, and patient goals. A morphology- and physiology-informed approach, rather than a technique-driven paradigm, offers the most rational path toward optimizing outcomes in this increasingly common geriatric injury.

## Supplementary Information

Below is the link to the electronic supplementary material.

Supplementary Material (PDF 2952 KB)

## Data Availability

No datasets were generated or analysed during the current study.

## Abbreviations

ASA: American Society of Anesthesiologists
ADS: Anterior Dens Screw
CINeMA: Confidence in Network Meta-Analysis
CI: Confidence intervals
CCI: Charlson Comorbidity Index
EQ-5D: EuroQol 5
ICU: Intensive Care Unit
NDI: Neck Disability Index
NMA: Network Meta-Analysis
NSM: Non-Surgical Management
OR: Odds Ratio
PA: Posterior Arthrodesis
PROM: Patient Reported Outcome Measure
ROBINS-I: Risk of Bias in Non-Randomized Studies of—Interventions tool
SF-36: Short Form 36 Health Survey
