# Supplementary material for: Determining the optimal management of geriatric type II odontoid fractures: a comparative network meta-analysis
Source: Neurosurg Rev. 2026 Mar 7;49(1):260. doi: 10.1007/s10143-026-04170-8 (PMC12967659; doi:10.1007/s10143-026-04170-8)
Supplement: Supplementary file 1 — Supplementary Material (PDF 2952 KB) [file 10143_2026_4170_MOESM1_ESM.pdf]

Database	Search strategy
**PubMed/MEDLINE (n=568)**	((odontoid OR dens) AND (fracture))<br><br>AND<br><br>((("anterior odontoid" OR "anterior screw" OR "anterior dens" OR "odontoid screw" OR "dens screw") AND ("posterior arthrodesis" OR "posterior fusion" OR "fixation" OR "atlanto-axial fusion" OR "C1-C2"))<br>OR<br>(("anterior odontoid" OR "anterior screw" OR "anterior dens" OR "odontoid screw" OR "dens screw") AND (nonoperative OR non-operative OR conservative OR "halo vest" OR "collar" OR "brace" OR "orthosis"))<br>OR<br>(("posterior arthrodesis" OR "posterior fusion" OR "fixation" OR "atlanto-axial fusion" OR "C1-C2") AND (nonoperative OR non-operative OR conservative OR "halo vest" OR "collar" OR "brace" OR "orthosis")))
**Embase (n=792)**	((odontoid OR dens) AND (fracture))<br><br>AND<br><br>((("anterior odontoid" OR "anterior screw" OR "anterior dens" OR "odontoid screw" OR "dens screw") AND ("posterior arthrodesis" OR "posterior fusion" OR fixation OR "atlanto-axial fusion" OR C1-C2))<br>OR<br>(("anterior odontoid" OR "anterior screw" OR "anterior dens" OR "odontoid screw" OR "dens screw") AND (nonoperative OR non-operative OR conservative OR "halo vest" OR collar OR brace OR orthosis))<br>OR<br>(("posterior arthrodesis" OR "posterior fusion" OR fixation OR "atlanto-axial fusion" OR C1-C2) AND (nonoperative OR non-operative OR conservative OR "halo vest" OR collar OR brace OR orthosis)))
**CENTRAL (n=10)**	((odontoid OR dens) AND fracture)<br><br>AND<br><br>((("anterior odontoid" OR "anterior screw" OR "anterior dens" OR "odontoid screw" OR "dens screw") AND ("posterior arthrodesis" OR "posterior fusion" OR fixation OR "atlanto-axial fusion" OR "C1-C2" OR "C1 C2" OR "C1–C2"))<br>OR<br>((nonoperative OR non-operative OR conservative OR "halo vest" OR collar OR brace OR orthosis) AND ("posterior arthrodesis" OR "posterior fusion" OR fixation OR "atlanto-axial fusion" OR "C1-C2" OR "C1 C2" OR "C1–C2"))<br>OR

	(("anterior odontoid" OR "anterior screw" OR "anterior dens" OR "odontoid screw" OR "dens screw") AND (nonoperative OR non-operative OR conservative OR "halo vest" OR collar OR brace OR orthosis)))

Supplementary Table 1: Search Strategies, n = number of articles returned

Risk of bias domains

Study	D1	D2	D3	D4	D5	D6	D7	Overall
Allia 2019	X	-	+	+	+	+	+	X
Andersson 2000	-	+	-	+	+	+	+	-
Chaudhary 2010	X	+	+	+	+	+	+	X
France 2012	-	+	+	+	+	+	+	-
Hamrick 2023	X	+	+	+	-	+	+	X
Huybregts 2024	X	+	+	+	+	+	+	X
Joestl 2016	+	+	+	+	+	+	+	+
Jung 2023	X	+	+	+	+	+	+	X
Kuntz 2000	X	+	+	+	+	+	+	X
Molinari 2013	X	-	+	+	+	+	+	X
Moscolo 2021	-	+	+	+	+	+	+	-
Omeis 2009	X	+	+	+	+	+	+	X
Patterson 2017	-	+	+	+	+	+	+	-
Perry 2017	+	+	+	+	+	+	+	+
Platzer 2007	-	+	+	+	-	+	+	-
Przkora 2006	X	+	+	+	+	+	+	X
Reinhold 2011	X	+	+	+	+	+	+	X
Scheyerer 2013	+	+	+	+	+	+	+	+
Shousha 2019	-	+	+	+	+	+	+	-

Domains:
D1: Bias due to confounding.
D2: Bias due to selection of participants.
D3: Bias in classification of interventions.
D4: Bias due to deviations from intended interventions.
D5: Bias due to missing data.
D6: Bias in measurement of outcomes.
D7: Bias in selection of the reported result.

Judgement
X Serious
- Moderate
+ Low

Supplementary Table 2: Risk of bias summary for non-randomized studies (ROBINS-I)

PRISMA NMA Checklist of Items to Include When Reporting A Systematic Review Involving a Network Meta-analysis

Section/Topic	Item #	Checklist Item	Reported on Page #
**TITLE**			
Title	1	Identify the report as a systematic review *incorporating a network meta-analysis (or related form of meta-analysis).*	1
**ABSTRACT**			2
Structured summary	2	Provide a structured summary including, as applicable: **Background:** main objectives **Methods:** data sources; study eligibility criteria, participants, and interventions; study appraisal; and *synthesis methods, such as network meta-analysis.* **Results:** number of studies and participants identified; summary estimates with corresponding confidence/credible intervals; *treatment rankings may also be discussed. Authors may choose to summarize pairwise comparisons against a chosen treatment included in their analyses for brevity.* **Discussion/Conclusions:** limitations; conclusions and implications of findings. **Other:** primary source of funding; systematic review registration number with registry name.	
**INTRODUCTION**			
Rationale	3	Describe the rationale for the review in the context of what is already known, *including mention of why a network meta-analysis has been conducted.*	3
Objectives	4	Provide an explicit statement of questions being addressed, with reference to participants, interventions, comparisons, outcomes, and study design (PICOS).	4
**METHODS**			
Protocol and registration	5	Indicate whether a review protocol exists and if and where it can be accessed (e.g., Web address); and, if available, provide registration information, including registration number.	5

Eligibility criteria	6	Specify study characteristics (e.g., PICOS, length of follow-up) and report characteristics (e.g., years considered, language, publication status) used as criteria for eligibility, giving rationale. *Clearly describe eligible treatments included in the treatment network, and note whether any have been clustered or merged into the same node (with justification).*	5
Information sources	7	Describe all information sources (e.g., databases with dates of coverage, contact with study authors to identify additional studies) in the search and date last searched.	5
Search	8	Present full electronic search strategy for at least one database, including any limits used, such that it could be repeated.	5
Study selection	9	State the process for selecting studies (i.e., screening, eligibility, included in systematic review, and, if applicable, included in the meta-analysis).	5
Data collection process	10	Describe method of data extraction from reports (e.g., piloted forms, independently, in duplicate) and any processes for obtaining and confirming data from investigators.	5-6
Data items	11	List and define all variables for which data were sought (e.g., PICOS, funding sources) and any assumptions and simplifications made.	6
**Geometry of the network**	**S1**	Describe methods used to explore the geometry of the treatment network under study and potential biases related to it. This should include how the evidence base has been graphically summarized for presentation, and what characteristics were compiled and used to describe the evidence base to readers.	6
Risk of bias within individual studies	12	Describe methods used for assessing risk of bias of individual studies (including specification of whether this was done at the study or outcome level), and how this information is to be used in any data synthesis.	6
Summary measures	13	State the principal summary measures (e.g., risk ratio, difference in means). *Also describe the use of additional summary measures assessed, such as treatment rankings and surface under the cumulative ranking curve (SUCRA) values, as well as modified approaches used to present summary findings from meta-analyses.*	6
Planned methods of analysis	14	Describe the methods of handling data and combining results of studies for each network meta-analysis. This should include, but not be limited to:<br>· *Handling of multi-arm trials;*<br>· *Selection of variance structure;*<br>· *Selection of prior distributions in Bayesian analyses; and*<br>· *Assessment of model fit.*	6

**Assessment of Inconsistency**	**S2**	Describe the statistical methods used to evaluate the agreement of direct and indirect evidence in the treatment network(s) studied. Describe efforts taken to address its presence when found.	6
Risk of bias across studies	15	Specify any assessment of risk of bias that may affect the cumulative evidence (e.g., publication bias, selective reporting within studies).	7
Additional analyses	16	Describe methods of additional analyses if done, indicating which were pre-specified. This may include, but not be limited to, the following:<br>· Sensitivity or subgroup analyses;<br>· Meta-regression analyses;<br>· *Alternative formulations of the treatment network; and*<br>· *Use of alternative prior distributions for Bayesian analyses (if applicable).*	6
**RESULTS†**			
Study selection	17	Give numbers of studies screened, assessed for eligibility, and included in the review, with reasons for exclusions at each stage, ideally with a flow diagram.	Fig. 1
**Presentation of network structure**	**S3**	Provide a network graph of the included studies to enable visualization of the geometry of the treatment network.	Fig. 2,3,4,5
**Summary of network geometry**	**S4**	Provide a brief overview of characteristics of the treatment network. This may include commentary on the abundance of trials and randomized patients for the different interventions and pairwise comparisons in the network, gaps of evidence in the treatment network, and potential biases reflected by the network structure.	8
Study characteristics	18	For each study, present characteristics for which data were extracted (e.g., study size, PICOS, follow-up period) and provide the citations.	8
Risk of bias within studies	19	Present data on risk of bias of each study and, if available, any outcome level assessment.	8
Results of individual studies	20	For all outcomes considered (benefits or harms), present, for each study: 1) simple summary data for each intervention group, and 2) effect estimates and confidence intervals. *Modified approaches may be needed to deal with information from larger networks.*	8

Synthesis of results	21	Present results of each meta-analysis done, including confidence/credible intervals. *In larger networks, authors may focus on comparisons versus a particular comparator (e.g. placebo or standard care), with full findings presented in an appendix. League tables and forest plots may be considered to summarize pairwise comparisons.* If additional summary measures were explored (such as treatment rankings), these should also be presented.	Fig. 2,3,4,5
**Exploration for inconsistency**	**S5**	Describe results from investigations of inconsistency. This may include such information as measures of model fit to compare consistency and inconsistency models, *P* values from statistical tests, or summary of inconsistency estimates from different parts of the treatment network.	9
Risk of bias across studies	22	Present results of any assessment of risk of bias across studies for the evidence base being studied.	Sup. Table 2
Results of additional analyses	23	Give results of additional analyses, if done (e.g., sensitivity or subgroup analyses, meta-regression analyses*, alternative network geometries studied, alternative choice of prior distributions for Bayesian analyses,* and so forth).	8
**DISCUSSION**			
Summary of evidence	24	Summarize the main findings, including the strength of evidence for each main outcome; consider their relevance to key groups (e.g., healthcare providers, users, and policy-makers).	11
Limitations	25	Discuss limitations at study and outcome level (e.g., risk of bias), and at review level (e.g., incomplete retrieval of identified research, reporting bias). *Comment on the validity of the assumptions, such as transitivity and consistency. Comment on any concerns regarding network geometry (e.g., avoidance of certain comparisons).*	14
Conclusions	26	Provide a general interpretation of the results in the context of other evidence, and implications for future research.	15
**FUNDING**			
Funding	27	Describe sources of funding for the systematic review and other support (e.g., supply of data); role of funders for the systematic review. This should also include information regarding whether funding has been received from manufacturers of treatments in the network and/or whether some of the authors are content	15

		experts with professional conflicts of interest that could affect use of treatments in the network.	

PICOS = population, intervention, comparators, outcomes, study design.
* Text in italics indicates wording specific to reporting of network meta-analyses that has been added to guidance from the PRISMA statement.
† Authors may wish to plan for use of appendices to present all relevant information in full detail for items in this section.

Supplementary Table 3: PRISMA-NMA checklist

Outcome	NSM n/N (%)	ADS n/N (%)	PA n/N (%)
**Mortality**	118/360 (32.8%)	28/300 (9.3%)	45/346 (13.0%)
**Union**	154/287 (53.7%)	180/253 (71.1%)	205/244 (84.0%)
**Unstable non-union**	55/241 (22.8%)	48/234 (20.5%)	28/213 (13.1%)
**Mechanical complications**	48/167 (28.7%)	4/104 (3.8%)	1/71 (1.4%)
**Systemic morbidity**	11/270 (4.1%)	33/232 (14.2%)	15/225 (6.7%)
**Secondary operation**	53/252 (21.0%)	59/260 (22.7%)	62/284 (21.8%)

Supplementary Table 4: Absolute event proportions pooled by treatment strategy. Note: These are pooled crude proportions across included studies and are provided for descriptive context only; comparative inferences are derived from the network meta-analysis. Abbreviations: ADS, anterior dens screw; NSM, non-surgical management; PA, posterior arthrodesis.

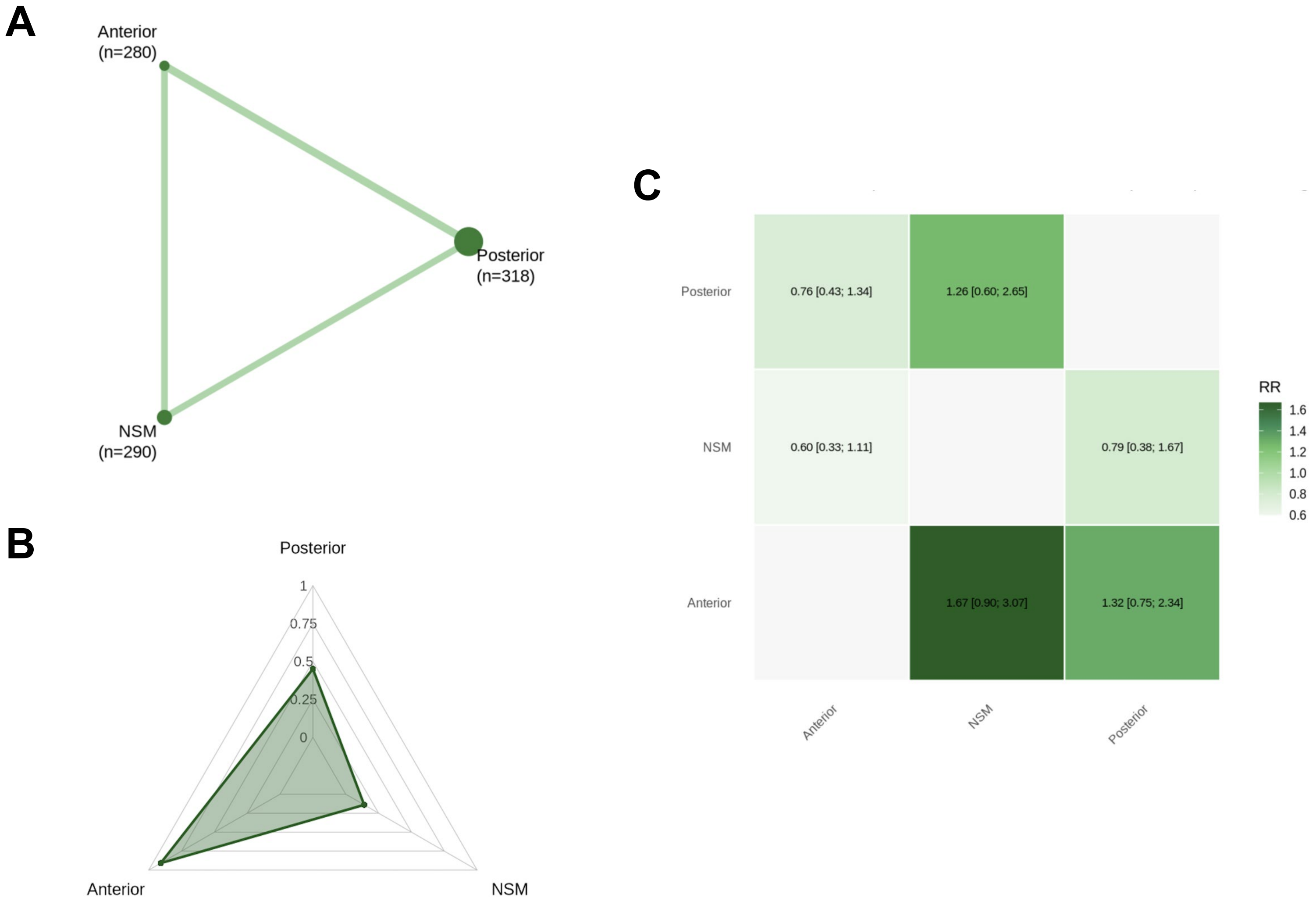

Supplementary Figure 1: Network meta-analysis for mechanical complications. (A) Network plot showing direct comparisons between interventions. (B) P scores radar plot (C) League table heatmap of intervention effects. Abbreviations: Anterior = Anterior Dens Screw Fixation. Posterior = Posterior Arthrodesis. NSM = Non-Surgical Management

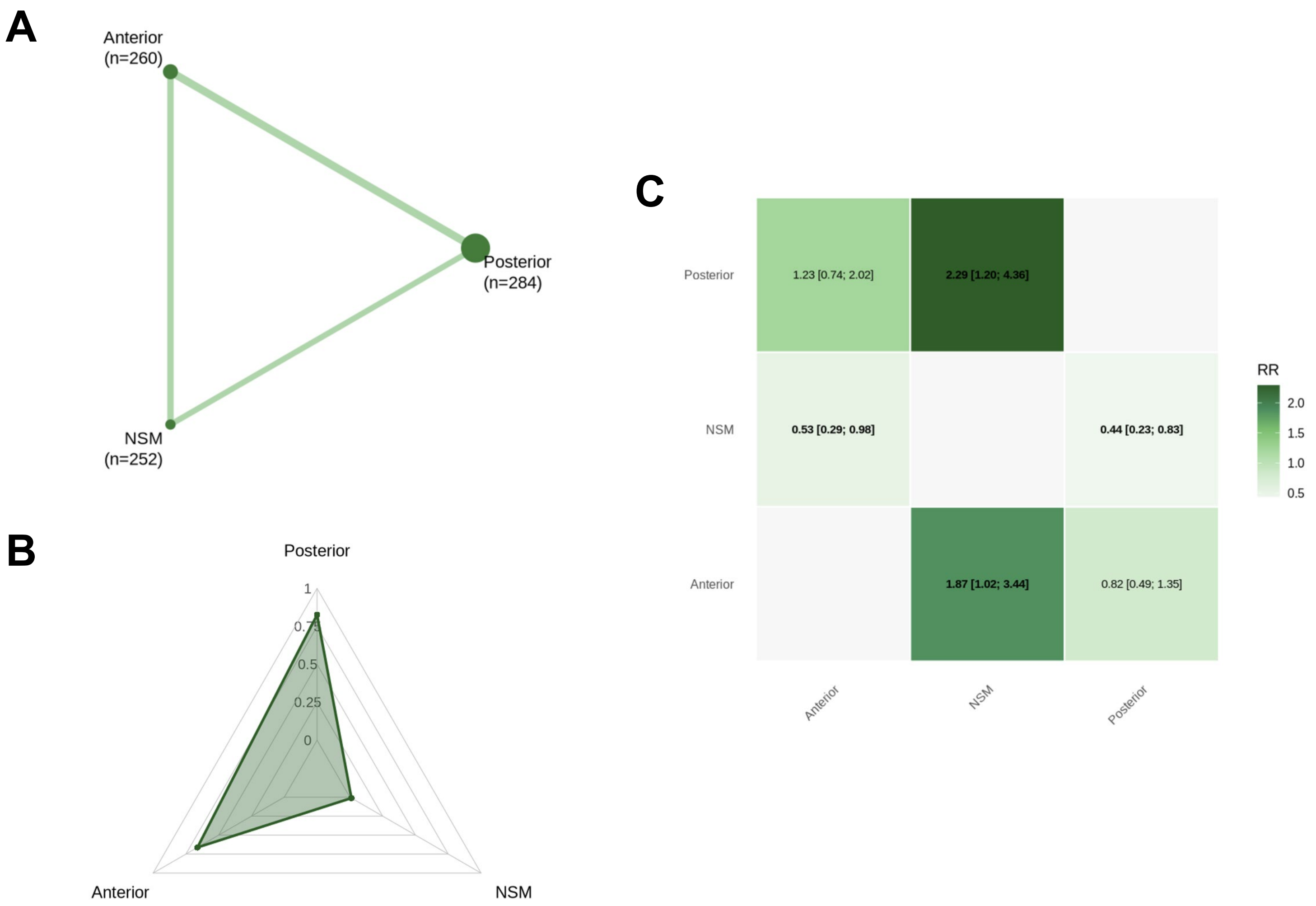

Supplementary Figure 2: Network meta-analysis for systemic morbidity. (A) Network plot showing direct comparisons between interventions. (B) P scores radar plot (C) League table heatmap of intervention effects. Abbreviations: Anterior = Anterior Dens Screw Fixation. Posterior = Posterior Arthrodesis. NSM = Non-Surgical Management

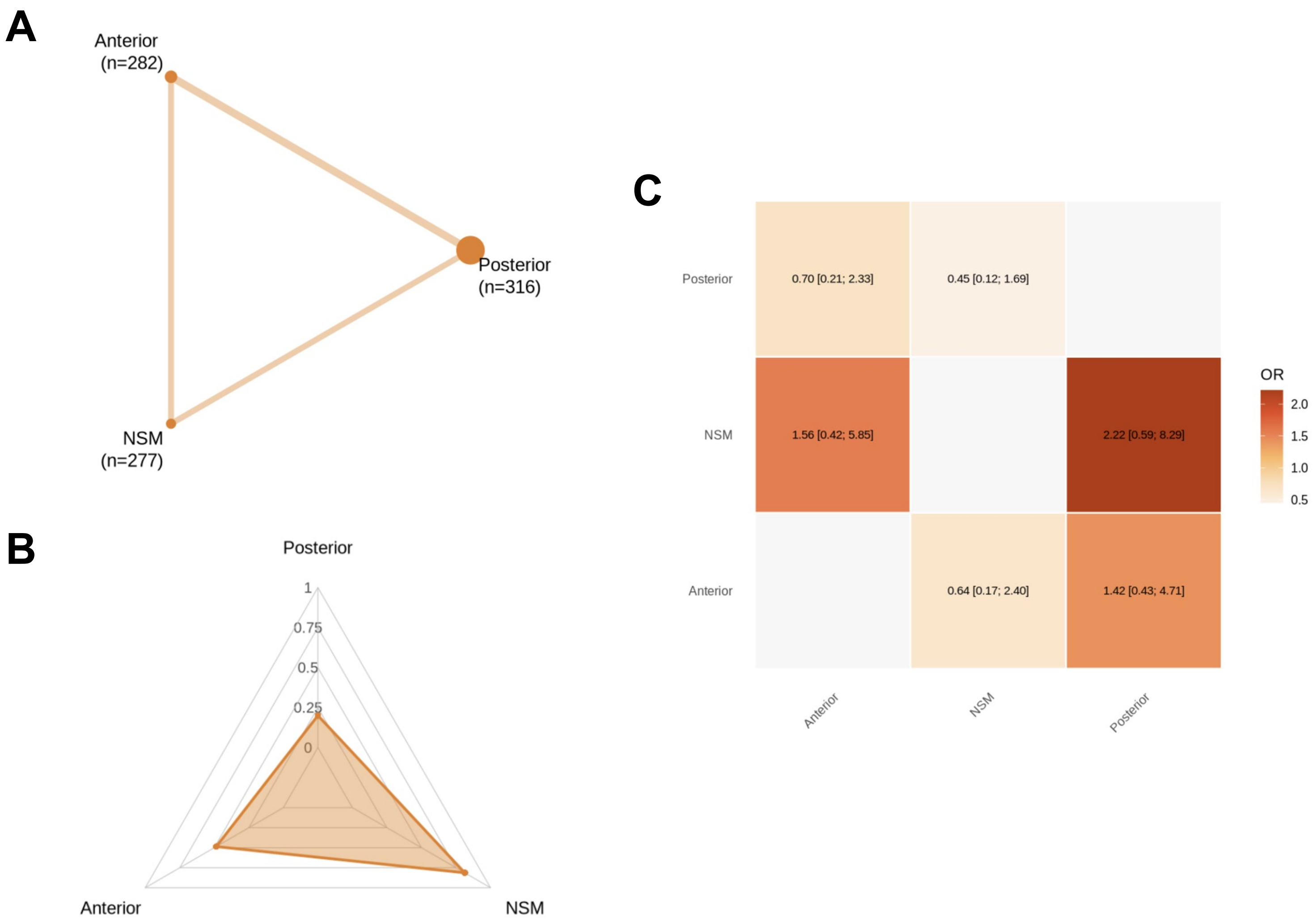

Supplementary Figure 3: Network meta-analysis for secondary operation. (A) Network plot showing direct comparisons between interventions. (B) P scores radar plot (C) League table heatmap of intervention effects. Abbreviations: Anterior = Anterior Dens Screw Fixation. Posterior = Posterior Arthrodesis. NSM = Non-Surgical Management

Supplementary Figure 4: Ranking probabilities (rankograms) for primary and secondary outcomes according to the interventional strategy. Abbreviations: Anterior = Anterior Dens Screw Fixation. Posterior = Posterior Arthrodesis. NSM = Non-Surgical Management

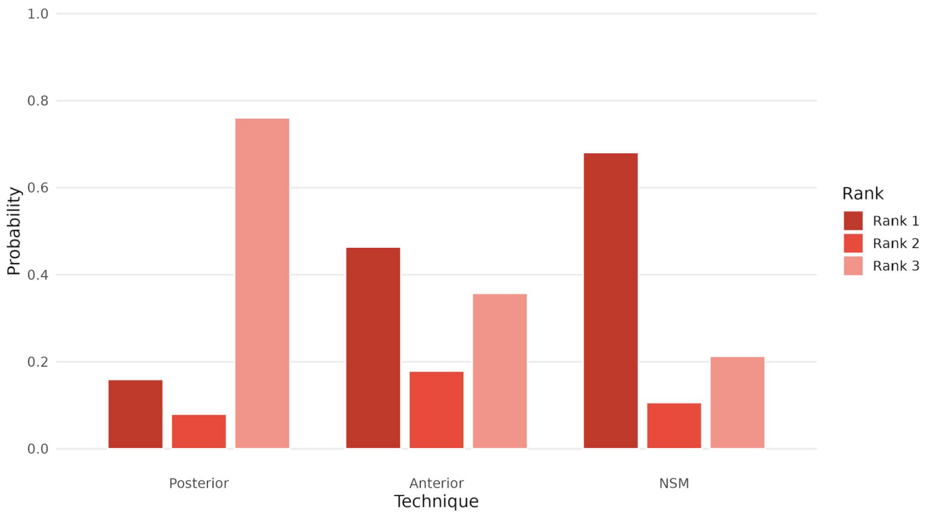

Supplementary Figure 4A: Ranking probabilities for mortality

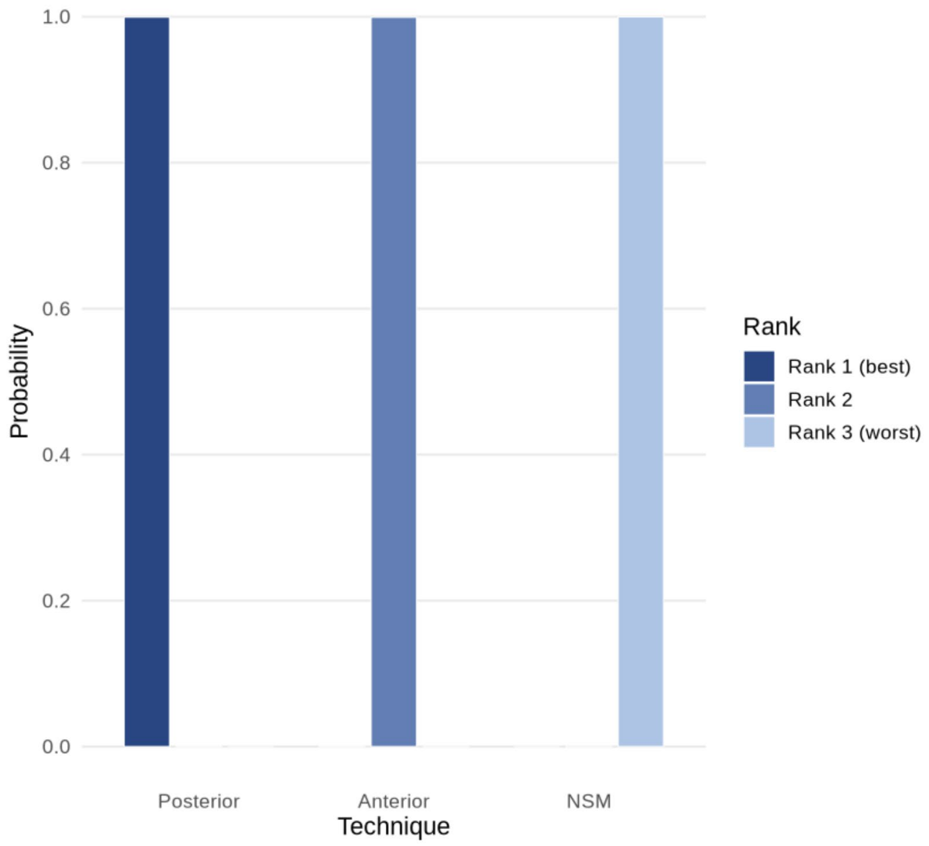

Supplementary Figure 4B: Ranking probabilities for union

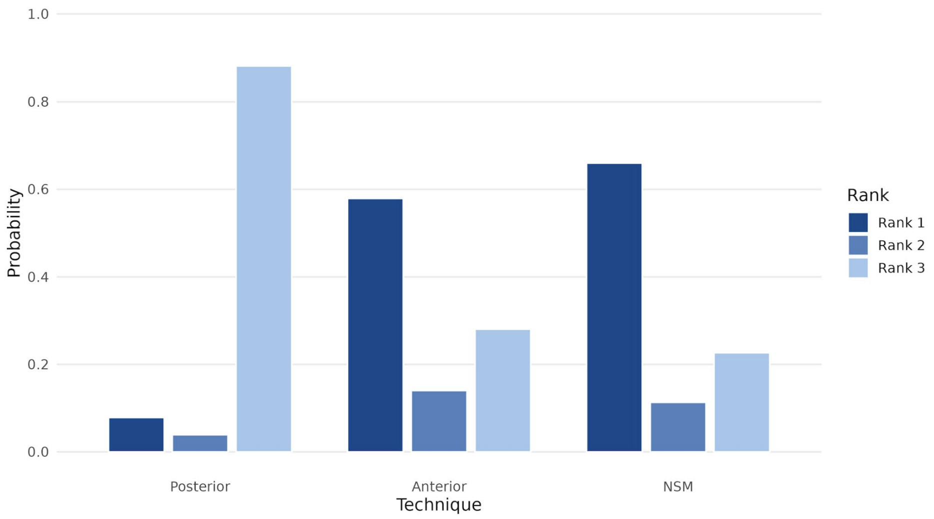

Supplementary Figure 4C: Ranking probabilities for stable non-union

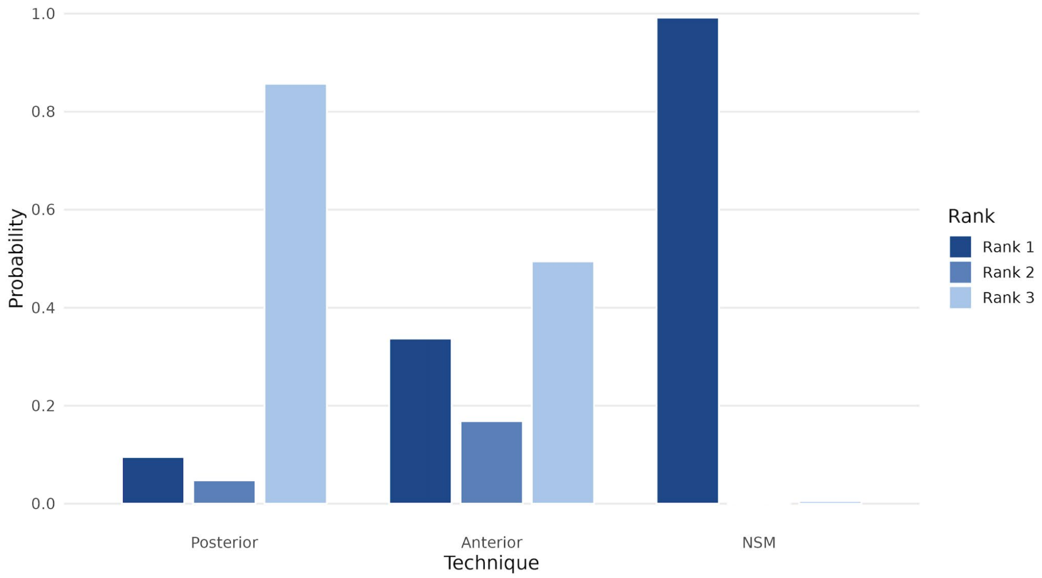

Supplementary Figure 4D: Ranking probabilities for unstable non-union

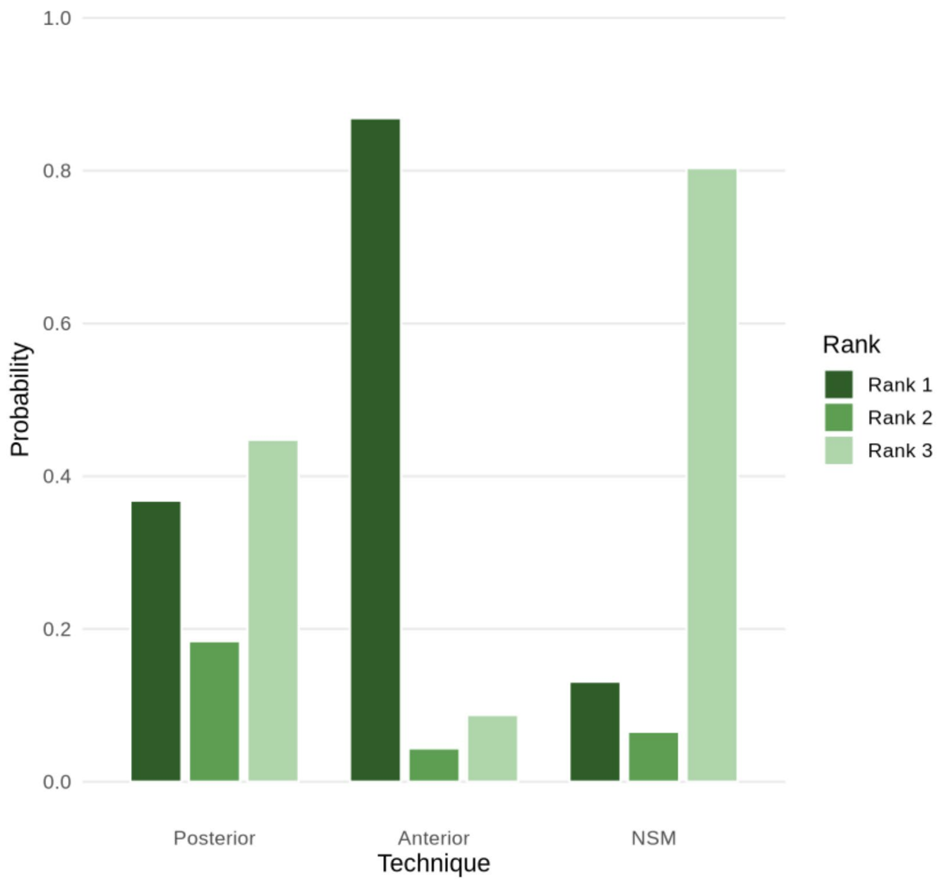

Supplementary Figure 4E: Ranking probabilities for mechanical complications

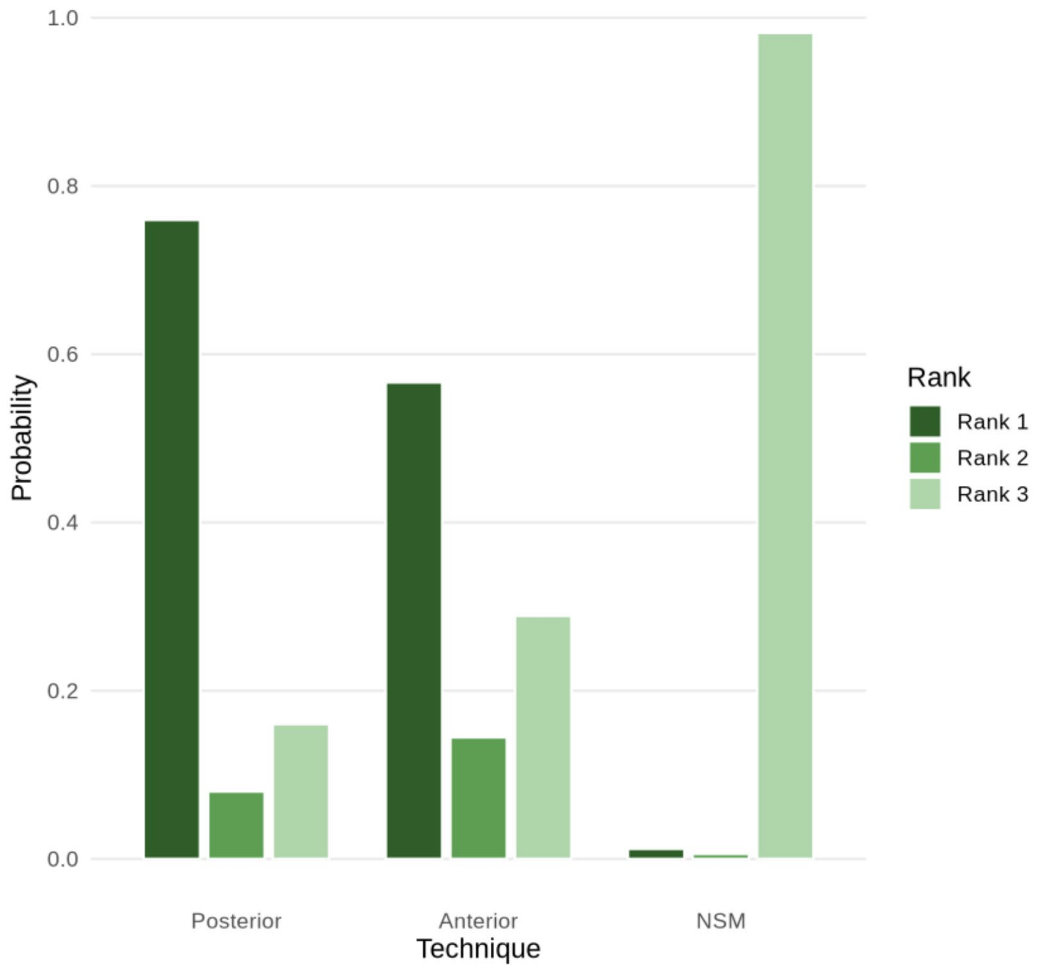

Supplementary Figure 4F: Ranking probabilities for systemic morbidity

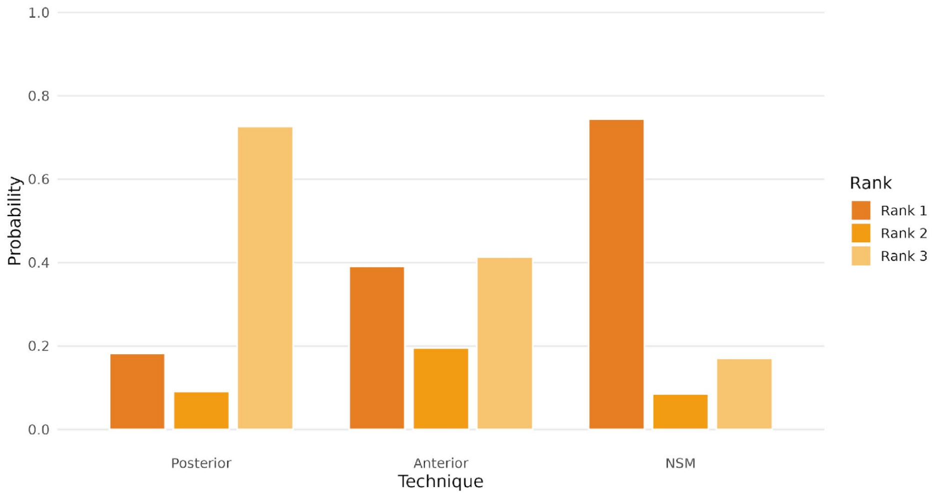

Supplementary Figure 4G: Ranking probabilities for secondary operation

Supplementary Figure 5: Node-split plot for each pairwise comparison. Abbreviations: Anterior = Anterior Dens Screw Fixation. Posterior = Posterior Arthrodesis. NSM = Non-Surgical Management

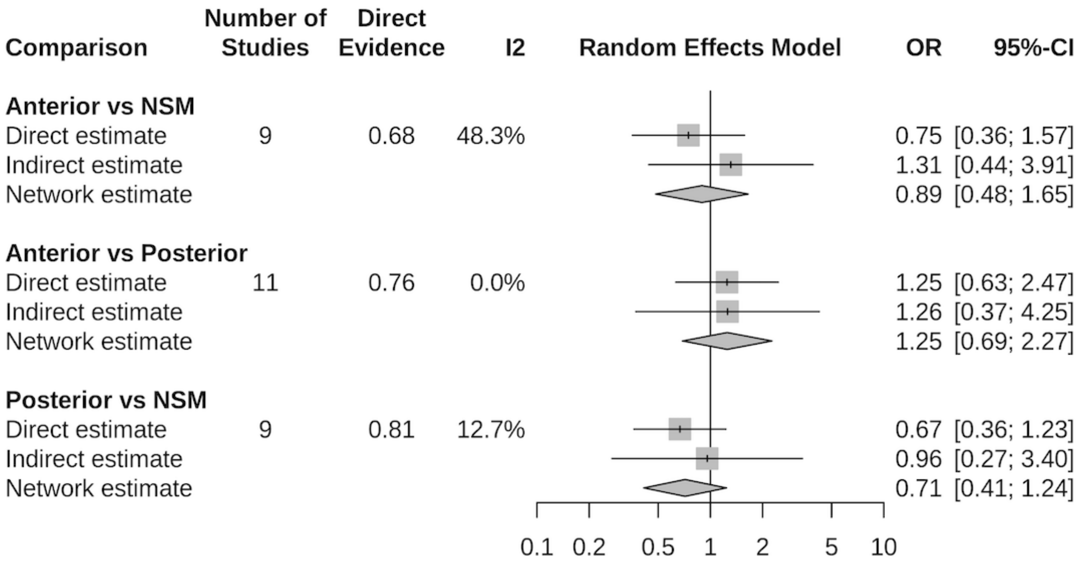

Supplementary Figure 5A: Node-split plot for mortality

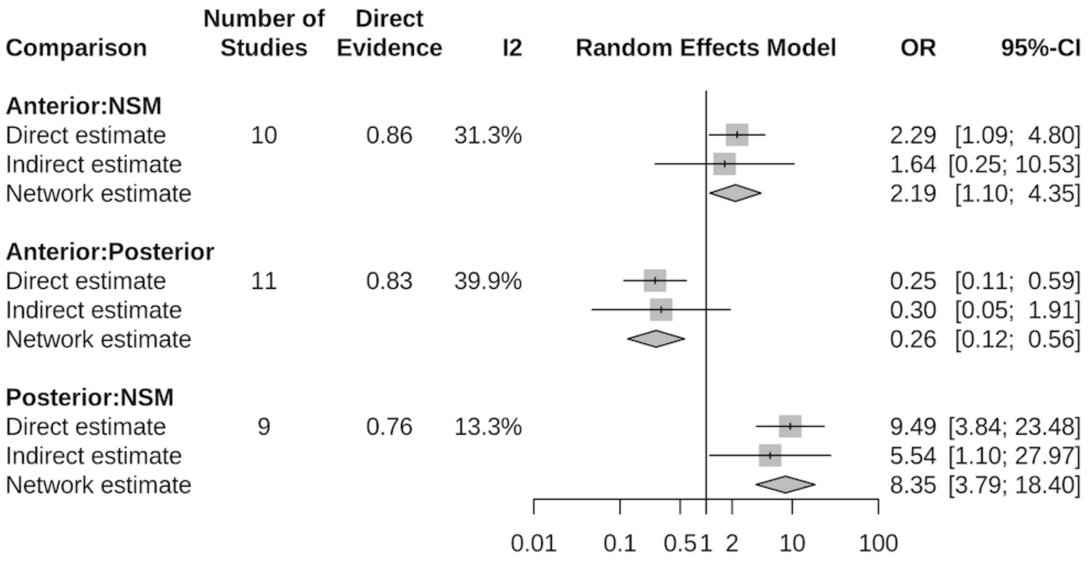

Supplementary Figure 5B: Node-split plot for union

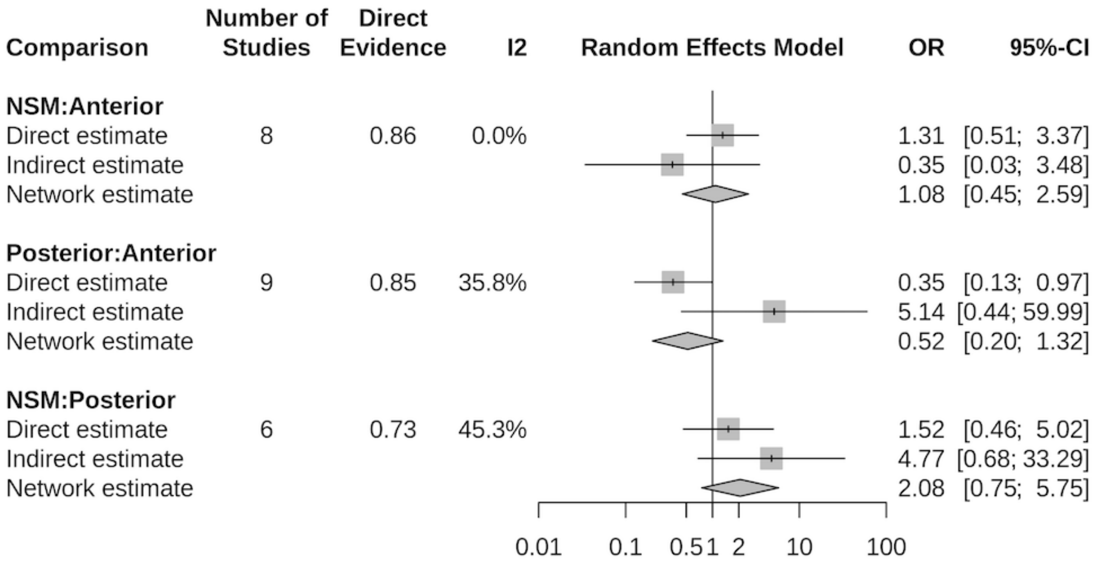

Supplementary Figure 5C: Node-split plot for stable non-union

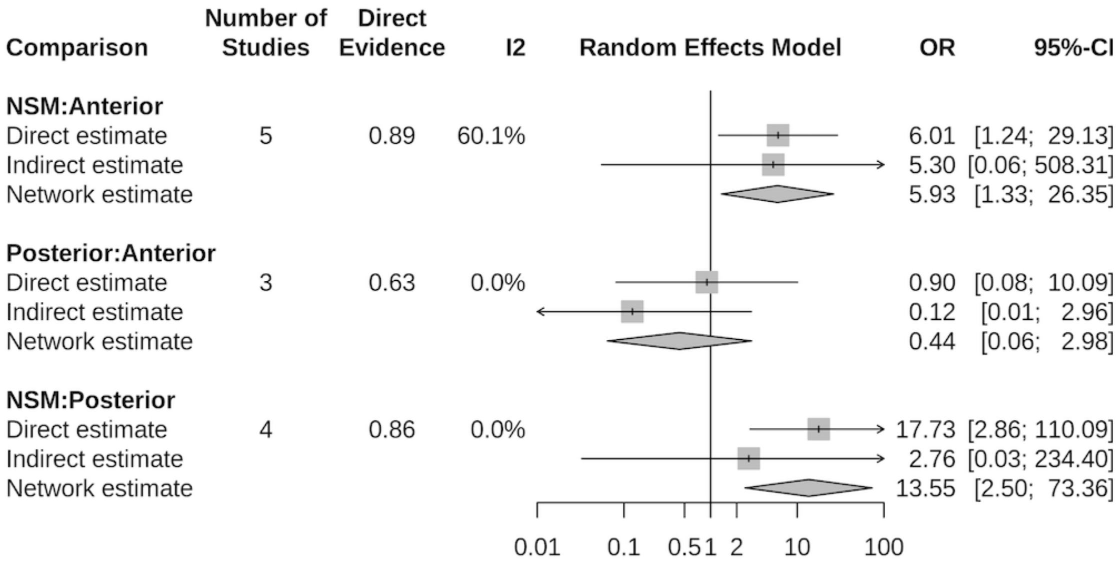

Supplementary Figure 5D: Node-split plot for unstable non-union

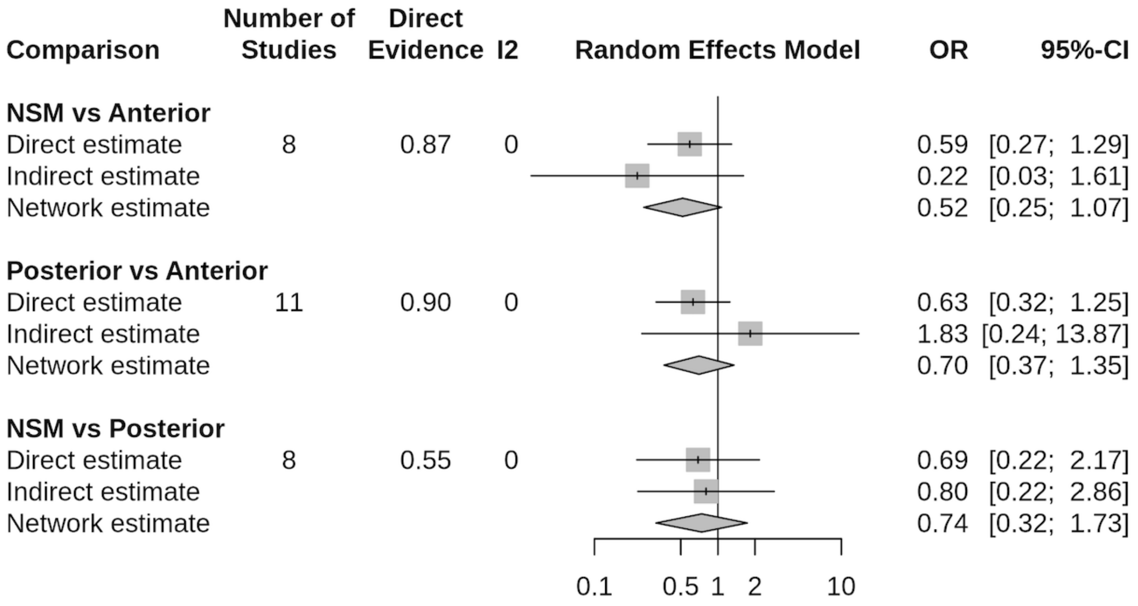

Supplementary Figure 5E: Node-split plot for mechanical complications

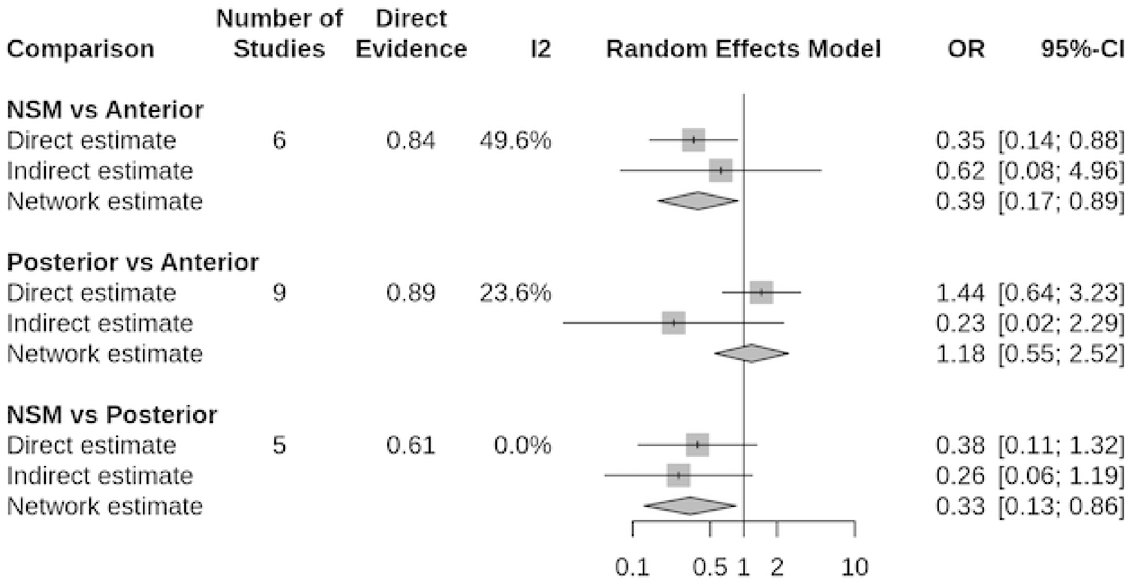

Supplementary Figure 5F: Node-split plot for systemic morbidity

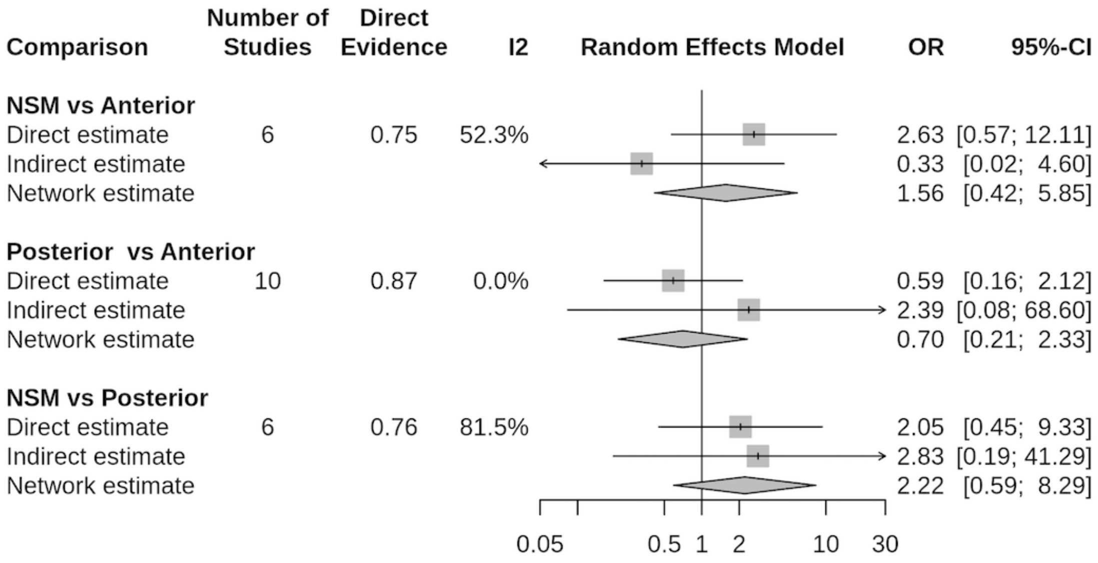

Supplementary Figure 5G: Node-split plot for secondary operation

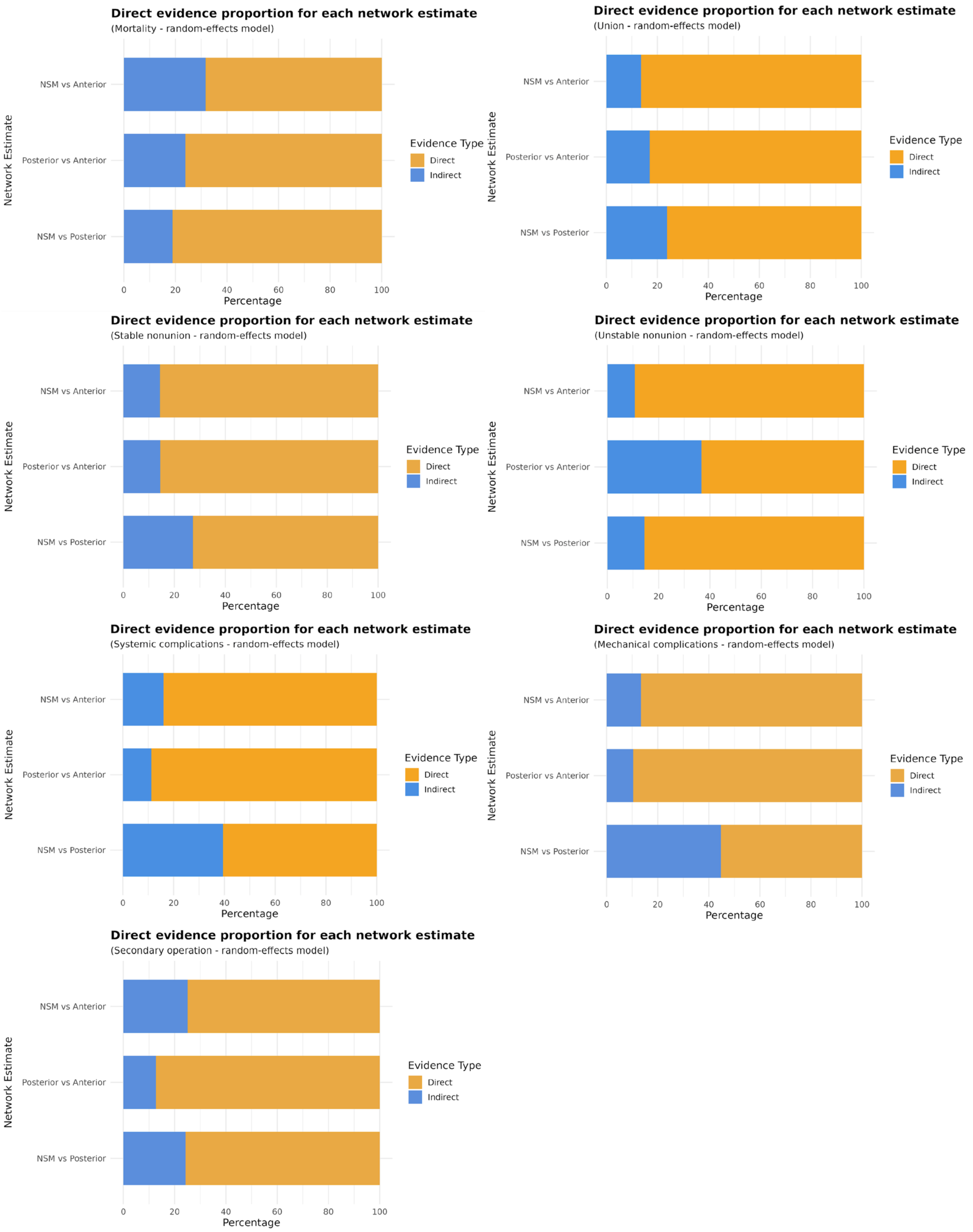

Supplementary Figure 6: Direct and indirect evidence proportion for each network estimate. Abbreviations: Anterior = Anterior Dens Screw Fixation. Posterior = Posterior Arthrodesis. NSM = Non-Surgical Management

Supplementary Figure 7: Publication Bias Assessment. Abbreviations: Anterior = Anterior Dens Screw Fixation. Posterior = Posterior Arthrodesis. NSM = Non-Surgical Management

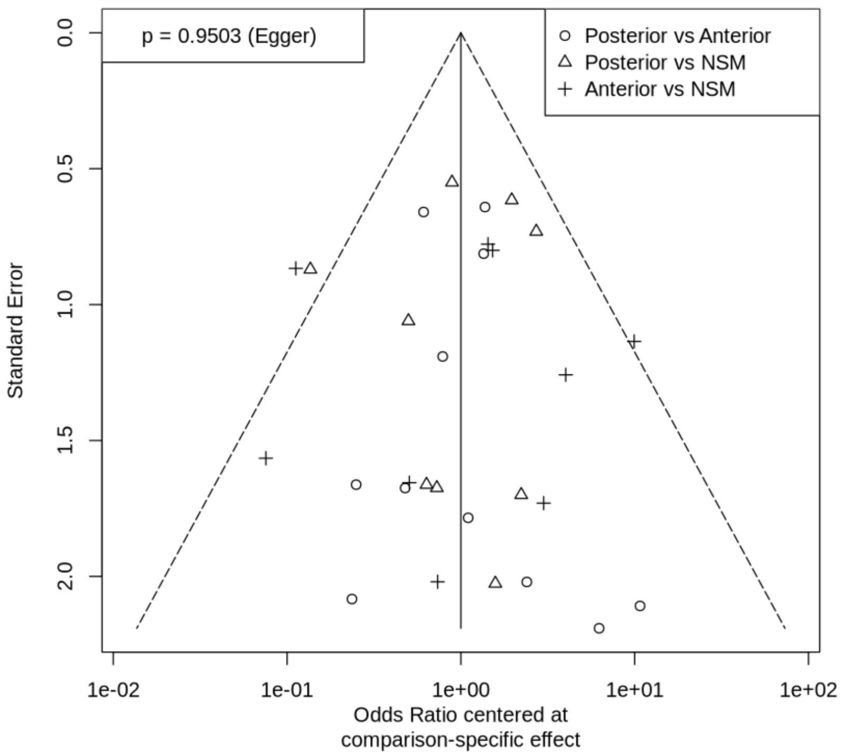

Supplementary Figure 7A: Funnel plot for mortality

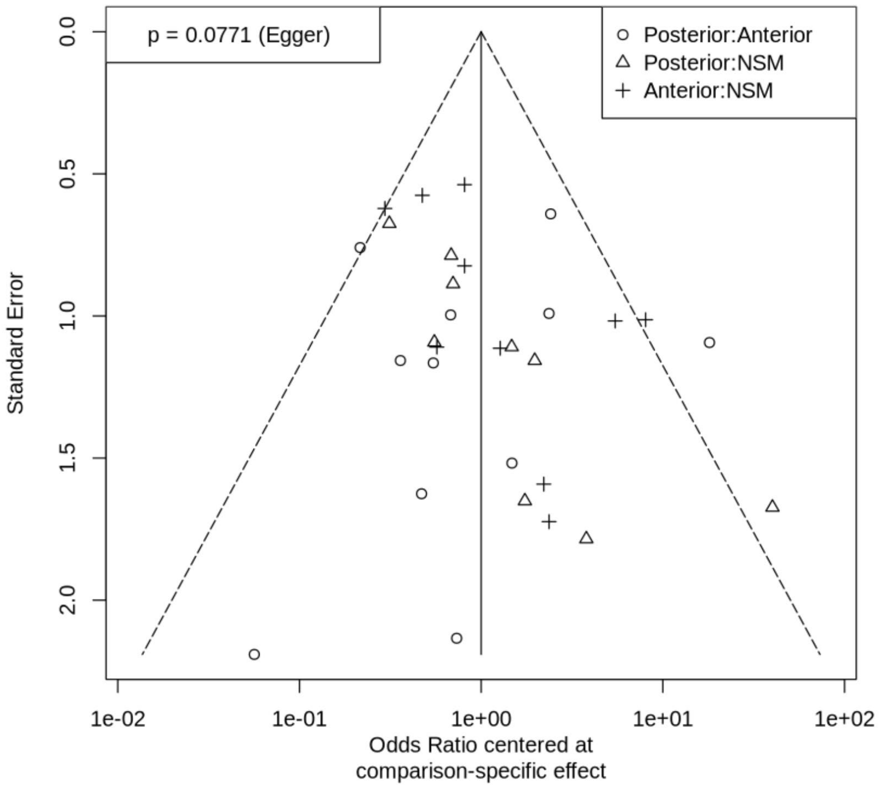

Supplementary Figure 7B: Funnel plot for union

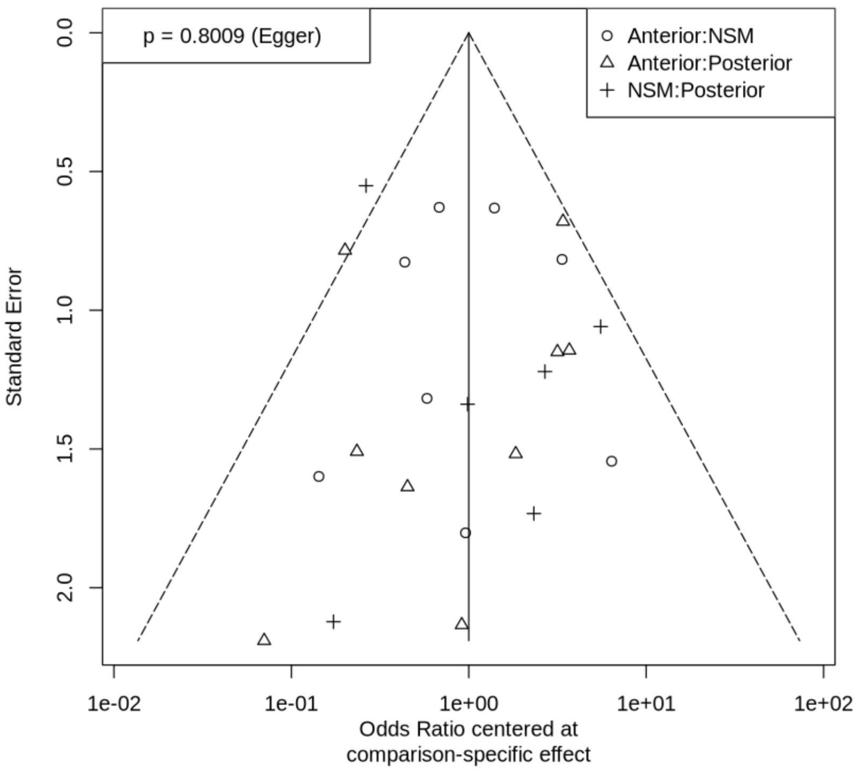

Supplementary Figure 7C: Funnel plot for stable non-union

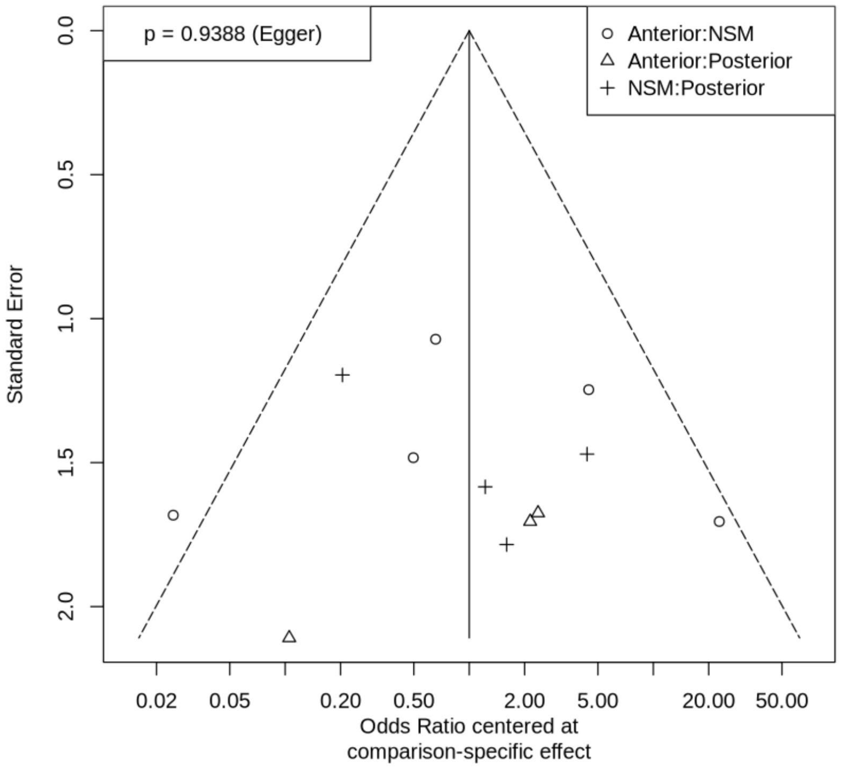

Supplementary Figure 7D: Funnel plot for unstable non-union

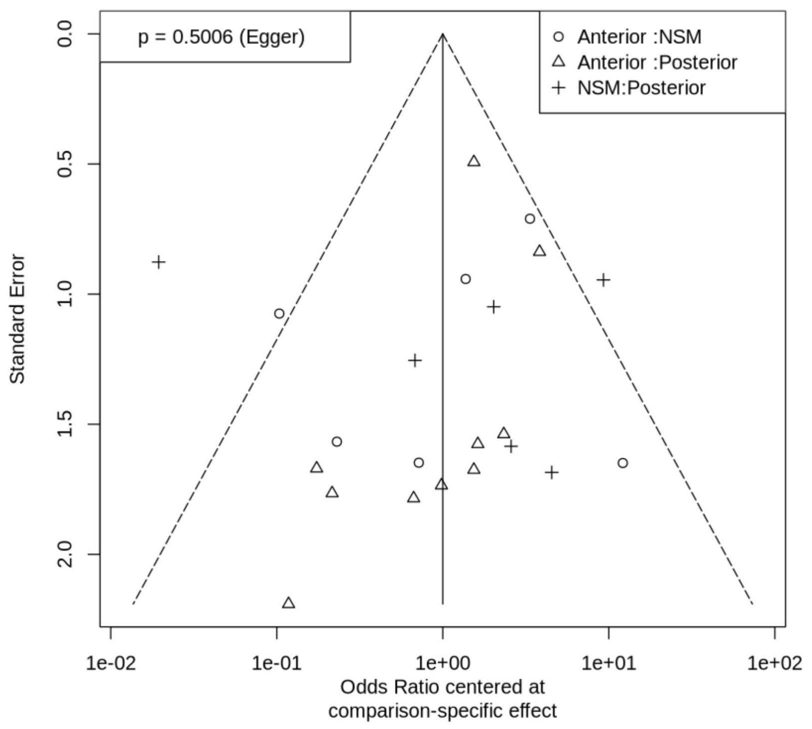

Supplementary Figure 7E: Funnel plot for secondary operation

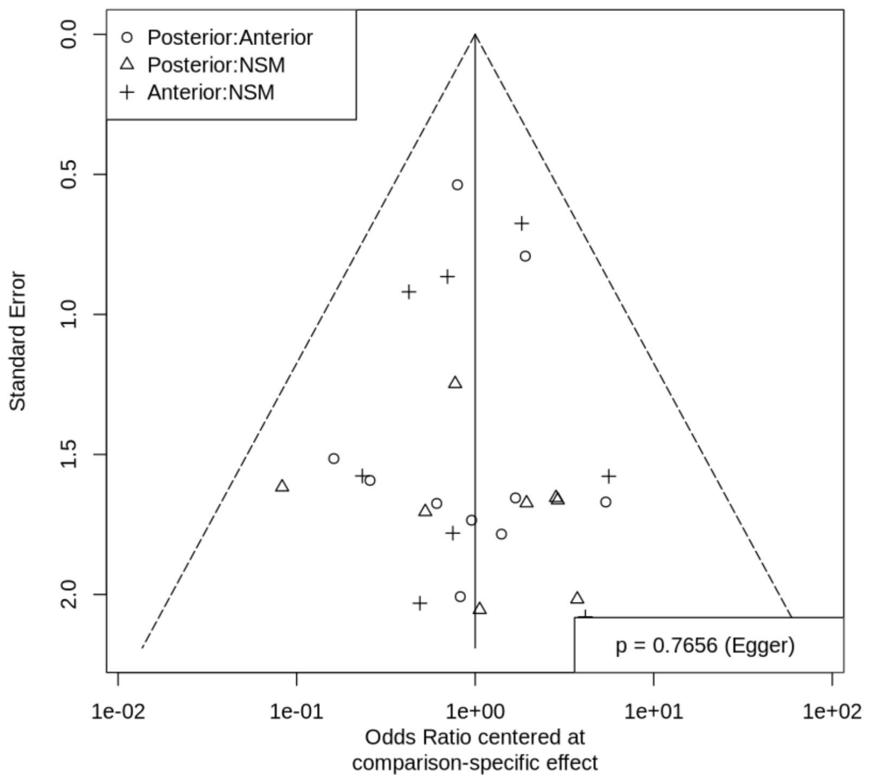

Supplementary Figure 7F: Funnel plot for mechanical complications

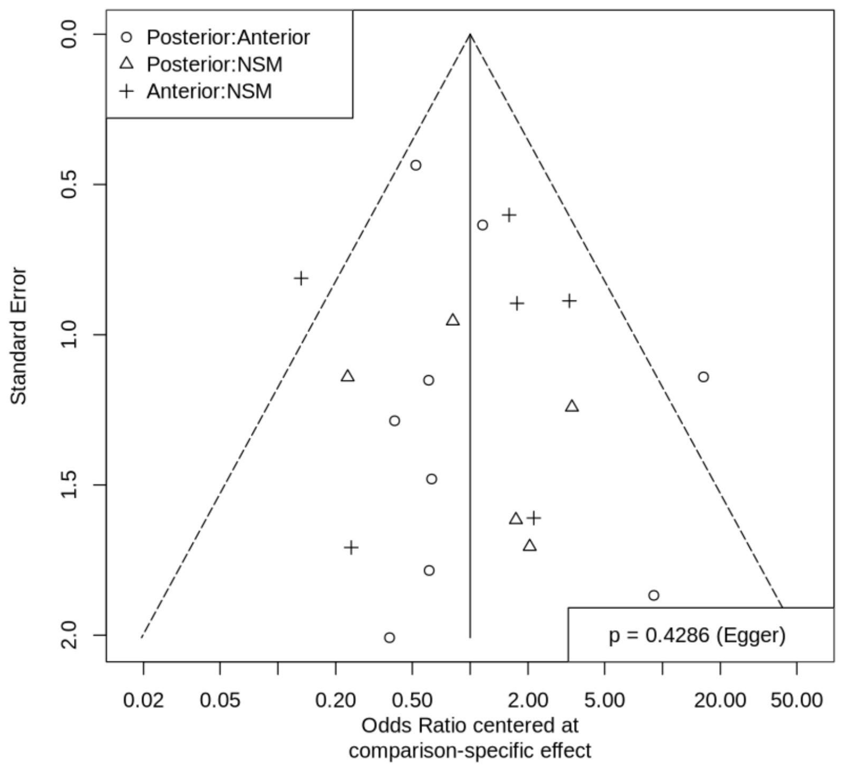

Supplementary Figure 7G: Funnel plot for systemic morbidity

Comparison	Number of Studies	Within-study bias	Reporting bias	Indirectness	Imprecision	Heterogeneity	Incoherence	Confidence rating	Reason(s) for downgrading
Mixed evidence									
**Anterior vs NSM**	9	Some concerns	Low risk	No concerns	Major concerns	No concerns	No concerns	High	| Within-study bias | Imprecision |
**Anterior vs Posterior**	11	Some concerns	Low risk	No concerns	Major concerns	No concerns	No concerns	High	| Within-study bias | Imprecision |
**NSM vs Posterior**	9	Some concerns	Low risk	No concerns	Major concerns	No concerns	No concerns	High	| Within-study bias | Imprecision |

Supplementary Figure 8: CINeMA plots
